# ZNF300 promotes chemoresistance and aggressive behaviour in non‐small‐cell lung cancer

**DOI:** 10.1111/cpr.12924

**Published:** 2020-10-19

**Authors:** Shilong Yu, Zhi Ao, Yi Wu, Liyuan Song, Peng Zhang, Xiaokang Li, Min Liu, Pin Qian, Ruijie Zhang, Xihua Li, Yan Chen, Xuanbin Wang, Xianhui Wang, Xuzhi Ruan, Guisheng Qian, Fuyun Ji

**Affiliations:** ^1^ Institute of Human Respiratory Disease Xinqiao Hospital the Army Medical University (Third Military Medical University) Chongqing China; ^2^ Department of Medical Biology School of Basic Medical Science Hubei University of Medicine Shiyan China; ^3^ Department of Respiratory Medicine The First Affiliated Hospital of Chongqing Medical University Chongqing China; ^4^ Taihe Hospital Hubei University of Medicine Shiyan China; ^5^ Department of Respiratory and Critical Care Medicine The Second Affiliated Hospital of Chongqing Medical University Chongqing China; ^6^ Hubei Key Laboratory of Wudang Local Chinese Medicine Research Biomedical Research Institute Hubei University of Medicine Shiyan China; ^7^ Institute of Biomedical Research Hubei University of Medicine Shiyan China; ^8^ Hubei Key Laboratory of Embryonic Stem Cell Research School of Basic Medical Science Hubei University of Medicine Shiyan China

**Keywords:** cell cycle, chemoresistance, differentiation, NSCLC, ZNF300

## Abstract

**Objectives:**

Chemoresistance induced by cisplatin has become the major impediment to lung cancer chemotherapy. This study explored the potential chemoresistant genes and underlying mechanisms of chemoresistance in NSCLC.

**Materials and methods:**

Gene expression profile was integrated with DNA methylation profile to screen the candidate chemoresistant genes. Bioinformatic analysis and immunohistochemistry were used to analyse the association of a candidate gene with the characteristics of NSCLC patients. Recombinant lentivirus vectors were utilized to overexpress or silence candidate gene. Microarrays and immunoblotting were applied to explore the downstream targets of candidate gene. Xenograft models were established to validate the findings in vitro.

**Results:**

An increased ZNF300 expression was detected in three chemoresistant cell lines of NSCLC, and the higher expression of ZNF300 was associated with poor OS of NSCLC patients. Cells with upregulated ZNF300 presented chemoresistance and enhanced aggressive growth compared to cells with downregulated ZNF300. ZNF300 inhibited MAPK/ERK pathways and activated CDK1 through inhibiting WEE1 and MYT1 and modulating MYC/AURKA/BORA/PLK1 axis. ICA and ATRA improved the anti‐tumour effect of cisplatin on chemoresistant cells by inducing differentiation.

**Conclusions:**

ZNF300 promotes chemoresistance and aggressive behaviour of NSCLC through regulation of proliferation and differentiation by downregulating MAPK/ERK pathways and regulation of slow‐cycling phenotype via activating CDK1 by inhibiting WEE1/MYT1 and modulating MYC/AURKA/BORA/PLK1 axis. Cisplatin, combined with ATRA and ICA, might be beneficial in chemoresistant cases of NSCLC.

## Graphical abstract

ZNF300 mediates chemoresistance of NSCLC. ZNF300 promotes aggressive growth of tumour cells correlating to poor prognosis of patients with NSCLC. ZNF300 inhibits MAPK/ERK signalling pathway to suppress proliferation and differentiation of chemoresistant cells. ZNF300 activates CDK1 by inhibiting WEE1 and MYT1 and modulating MYC/AURKA/BORA/PLK1 axis to regulate cell cycle of chemoresistant cells. ICA and ATRA improves anti‐tumour effect of cisplatin on chemoresistant cells by inducing differentiation.

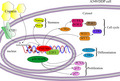

## 1 INTRODUCTION

Global Cancer Statistics 2018 demonstrates that lung cancer remains the most commonly diagnosed cancer and is the leading cause of cancer death in both sexes combined worldwide.^1^ Non‐small‐cell lung cancer (NSCLC) comprises approximately 80%‐85% of all lung cancers, with adenocarcinoma and squamous cell carcinoma being the predominant histological subtypes of NSCLC. Clinically, except for a small portion of NSCLC patients diagnosed at an early stage (stage I or II), over 60% of lung cancer patients present with locally advanced or metastatic disease (stage III or IV) at the time of diagnosis.

For NSCLC patients with advanced stages, the multiple therapeutic choices including molecular targeted therapy and immunotherapy have emerged and reached the clinic, leading to improved clinical outcomes in recent years.^2^, ^3^ However, the approved molecular targeted therapy or immunotherapy is limited to a small portion of NSCLC patients with advanced stages who possess the genetic alteration. Therefore, the platinum‐based chemotherapeutic agents continue to be the most widely prescribed chemotherapy for the vast majority of NSCLC patients, including those who failed in the genetic alteration‐guided targeted therapies and patients of stage I to IIIa who have undergone the complete surgical resection. Thus, the use of platinum agents, including cisplatin (DDP), remains unwavering despite chemoresistance associated with treatment failures.

Over the past three decades, intense research has been conducted and several mechanisms that account for the cisplatin‐resistant phenotype of tumour cells were explored.^4^ Though the known mechanisms explain the cisplatin resistance at the molecular level to a certain extent, cisplatin resistance often exhibits a multifactorial nature. One of the hard facts is that the micromolecules targeting the currently known mechanisms of chemoresistance have not achieved the expected effect on the tumour suppression in the related clinical researches having little or no impact on progression‐free survival (PFS) or overall survival (OS) of NSCLC.^5^ Moreover, clinically, once the tumour cells present the cisplatin‐resistant phenotype, the disease progresses malignantly and develops multidrug resistance to different chemotherapeutic agents with different pharmacological mechanisms, distant metastasis, and relapse, eventually leading to the treatment failure. Thus, there is an urgent need to continue exploring the mechanisms of chemoresistance in NSCLC.

In the study, the increased ZNF300 expression induced by cisplatin was found to be related to chemoresistance and malignant progression of NSCLC. Experiments in vitro and in vivo demonstrated that ZNF300 might suppress proliferation and differentiation via inhibiting MAPK/ERK signalling pathway and regulate slow‐cycling phenotype by activating CDK1 through inhibiting WEE1 and MYT1 and modulating MYC/AURKA/BORA/PLK1 axis to promote the chemoresistance and malignant progression of NSCLC. Cisplatin, combined with ATRA and ICA, especially ICA, might be beneficial for NSCLC patients who exhibited the chemoresistant phenotype.

## 2 MATERIALS AND METHODS

### 2.1 Cell lines and culture

Cell lines of human lung adenocarcinoma A549, H1650 and H1915; cell line of human lung squamous carcinoma H520; and cell line of human small cell lung cancer (SCLC) H446 were purchased from the Institute of Biochemistry and Biology, Chinese Academy of Sciences (Shanghai, China). The cisplatin‐resistant cell lines A549/DDP, H1650/DDP, H1915/DDP, H520/DDP and H446/DDP were established as described in our previous work.^6^, ^7^ All of the cells were tested for mycoplasma routinely. The identity and purity of cells were validated by short tandem repeat (STR) analysis profiling.

### 2.2 Lentivirus vectors and transfection

Four sets of ZNF300‐shRNA oligonucleotide were cloned into the LV3 lentiviral vectors (shZNF300) to silence ZNF300 in A549/DDP cells, whereas the full‐length sequence coding for human ZNF300 transcript variant 1 was cloned into the LV5 lentiviral vectors to overexpress ZNF300 in A549 cells. The sequences of ZNF300‐shRNA oligonucleotides are shown in Supplemental Materials and Experimental Procedures (Document S1).

### 2.3 Gene expression profiles

For processing on microarrays, total RNA (1‐2 µg) extracted using TRIzol and prepared using the 3'IVT Express Kit was utilized to synthesize double‐stranded cDNA, which was hybridized on the microarrays with probes for >54 000 genome‐wide transcripts. Microarray quality was verified by a signal intensity ratio of GAPDH 3′ to 5′ probe sets ≤ 3.0 and multi‐chip normalization scaling factor ≤ 10.0. Gene expression was normalized using the single‐channel array normalization (SCAN)/Universal exPression Codes (UPC) method. Genes with a UPC value of <0.2 in all samples were excluded.

### 2.4 Immunohistochemistry of clinic specimens and tissue microarray

Immunohistochemistry (IHC) was performed, and percentage of the positively staining cells was scored as described previously.^8^ Since the OS data of patients collected from Xinqiao Hospital were incomplete, a commercial tissue microarray containing 80 tumorous cases and 80 para‐carcinoma tissues (controls) with the OS data of lung adenocarcinoma patients were stained with ZNF300 antibody (Shanghai Zhuohao Medical Science and Technology Co. Ltd). The appraisal and statistics were completed as described above.

### 2.5 Co‐immunoprecipitation assay (Co‐IP)

The coding regions of ZNF300 were cloned into the ptt5 vector. A FLAG‐tag was fused at the N‐terminus of ZNF300. The recombinant vectors were then transformed into A549 cells to express ZNF300. The whole‐cell lysates were pre‐cleared and incubated with rabbit anti‐Flag antibody. The IP targets were disassociated from the immobilized antibodies and subjected to immunoblotting.

### 2.6 Video and imaging of co‐cultured cells

After seeded in 96‐well plate and cultured overnight, cells were subjected to the real‐time monitoring by High Content Analysis System (PerkinElmer Operetta CLS™) in fresh medium containing cisplatin (2 µg/mL), ATRA (5 µg/mL), ICA (20 µg/mL), separately or combined, for 7 days successively. Random vision fields were selected and transformed into videos. Pictures were captured at the end of each video.

### 2.7 Xenograft tumorigenic experiment

Healthy Balb/c‐nu mice (3‐ to 4‐week‐old female, n = 40) were purchased from Animal Laboratory of Beijing Vital River Laboratory Animal Technology Co. Ltd and housed in a climate‐control specific pathogen‐free (SPF) facility. Mice were divided into four groups that were inoculated subcutaneously with A549/DDP‐shZNF300‐NC, A549/DDP‐shZNF300, A549‐ZNF300‐NC and A549‐ZNF300 cells into the right flank (100 µL, 1 × 10^6^ cells/animal), respectively. Each group was then randomly divided into two subgroups and raised by professional breeders. Tumour volume was measured every 3 days from day 0. When the tumour volume reached 100 mm^3^, one subgroup in each group received an intraperitoneal injection with cisplatin (0.02 mg/10 g: cisplatin/animal weight) every 3 days while the other subgroup with normal saline as controls (NS). After 42 days, all animals were sacrificed by cervical dislocation, and xenograft tumours were excised. Tumour size and weight were recorded, and tumour volume (mm^3^) was calculated according to the formula *a* × *b* ^2^/2 (*a* = largest diameter; *b* = smallest diameter). The separated xenograft tumours were sectioned for haematoxylin and eosin (HE) staining, IHC and Western blotting. All procedures were carried out abiding by the Laboratory Animal‐Guideline for Ethical Review of Animal Welfare issued by the National Standard of the People's Republic of China and the Guiding Opinions on the Treatment of Laboratory Animals issued by the Ministry of Science and Technology of the People's Republic of China (GB/T35892‐2018).

### 2.8 Statistical analyses

The numerical data were expressed as means ± standard deviation. One‐way ANOVA was used to analyse the difference between means, and Pearson's chi‐square test or Fisher's exact test was used for categorical variables. The independent effect of ZNF300 was assessed by performing the multivariate logistic regression analysis with adjustments for the possible confounding factors of age, gender and smoking habit to calculate the adjusted *P‐*value. The Statistical Package for Social Science 15 for Windows was used for all statistical analyses (SPSS Inc). A *P‐*value <.05 was considered to be statistically significant. All experiments were performed a minimum of three times.

## 3 RESULTS

### 3.1 ZNF300 mediates chemoresistance of NSCLC

As shown in Figure S1A, IC_50_ of cisplatin, gemcitabine, paclitaxel, docetaxel and pemetrexed on A549/DDP cells was significantly higher than that of its progenitor A549 cells. Because mitochondrial apoptosis induction accounts for one of the primary mechanisms of cisplatin anti‐tumour activity, we compared the function changes of mitochondria between A549/DDP cells and A549 cell after treated with cisplatin. As shown in Figure S1B,E, mitochondrial functions of A549/DDP cells were less affected compared to A549 cells after treated with cisplatin. Similarly, H1650/DDP, H520/DDP, H1915/DDP and H446/DDP cells all presented the chemoresistant phenotype upon being tested with the same measurement (data not shown). Because A549 cells were widely used in the scientific studies of lung cancer, A549 and A549/DDP cells were utilized to screen the potential chemoresistant genes by HG‐U133 Plus 2.0 microarrays (Affymetrix). The microarray data have been uploaded to the GEO database with the accession number GSE154243 (https://www.ncbi.nlm.nih.gov/geo/). The GO enrichment and KEGG pathway classification (|FC|≥3) of the differentially expressed genes could be provided upon request.

**FIGURE 1:**
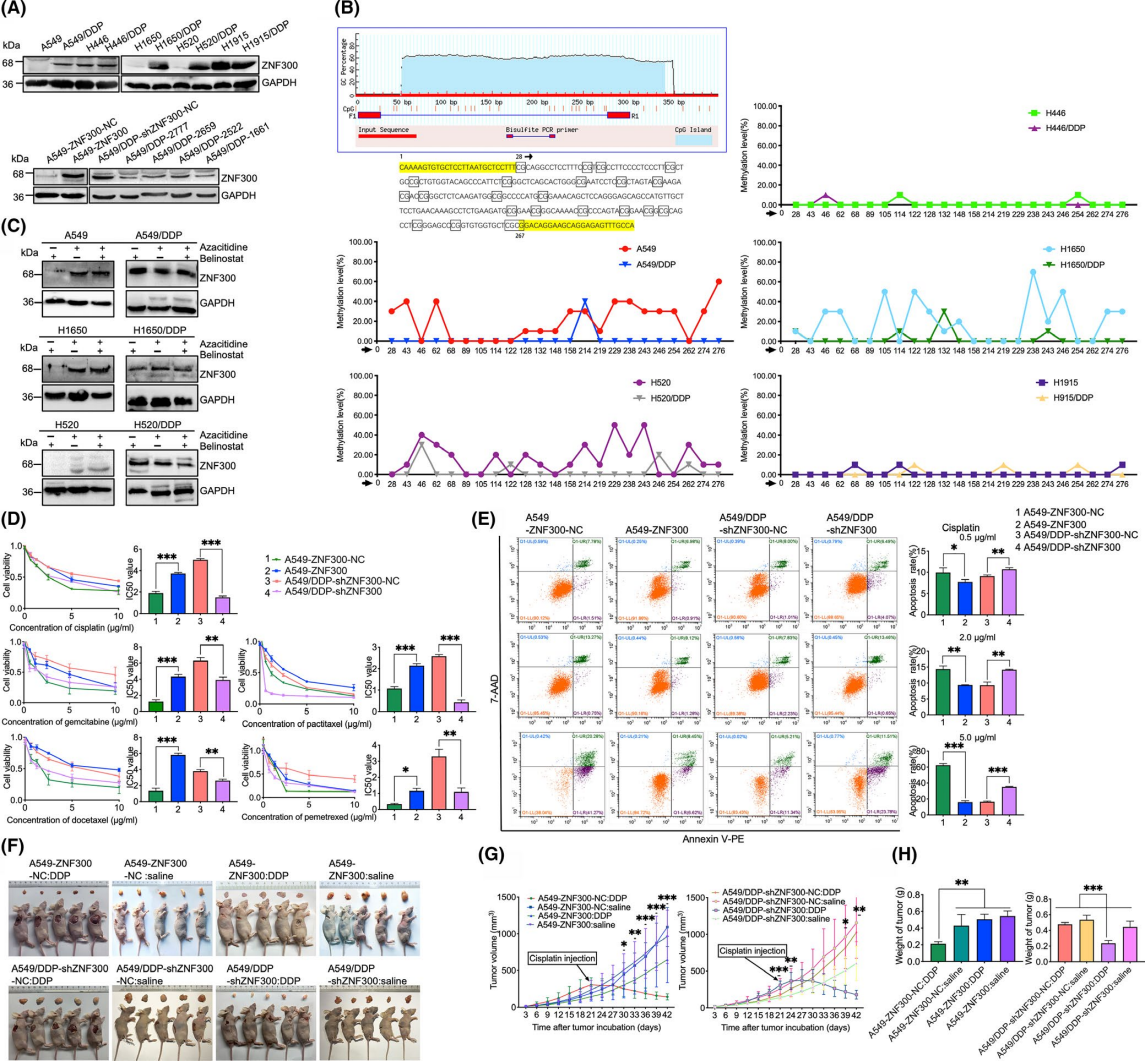
The upregulated ZNF300 induced by cisplatin promoted the chemoresistance in NSCLC. A, Expression of ZNF300 in the chemoresistant cells and their progenitor cells of human lung cancer (A549/DDP, A549, H1650/DDP, H1650, H1915/DDP and H1915: cell lines of human lung adenocarcinoma; H520/DDP and H520: cell lines of human lung squamous carcinoma; H446/DDP and H446: cell lines of human SCLC). B, Methylation status of the ZNF300 promoter in the cells mentioned above. C, Expression of ZNF300 in A549, A549/DDP, H1650, H1650/DDP, H520 and H520/DDP cells after treatment with azacitidine and belinostat, separately or combinedly. D, IC_50_ of cisplatin on A549‐ZNF300‐NC, A549‐ZNF300, A549/DDP‐shZNF300‐NC and A549/DDP‐shZNF300 cells, respectively. E, Apoptosis of A549‐ZNF300‐NC, A549‐ZNF300, A549/DDP‐shZNF300‐NC and A549/DDP‐shZNF300 cells after treatment with cisplatin. F, Observation of xenograft tumours in nude mice treated with cisplatin or normal saline. The nude mice were inoculated subcutaneously with A549‐ZNF300‐NC, A549‐ZNF300, A549/DDP‐shZNF300‐NC and A549/DDP‐shZNF300 cells into the right flank, respectively. G, Growth curves of xenograft tumours in each subgroup treated with cisplatin or normal saline. H, Measurement of the final tumour weight in each subgroup treated with cisplatin or normal saline. Data are expressed as the mean ± SD (significant difference between two groups: *P < .05, **P < .01, ***P < .001)

Integration of the expression profile (|FC|≥3) with the published MeDIP‐ChIP data^6^ revealed that 77 genes with promoter hypomethylation were upregulated, and 63 genes with promoter hypermethylation were downregulated in A549/DDP cells compared to A549 cells. RT‐PCR results confirmed that, out of the 140 genes, 15 genes with hypomethylated promoters were upregulated, while 25 genes with hypermethylated promoters downregulated in A549/DDP cells related to A549 cells (Figure S2). The primer sequences for RT‐PCR are presented in Table S1. Among the 15 upregulated genes, ZNF300 was selected as the candidate chemoresistant gene to be investigated comprehensively in the study because ZNF300 expression was negligible in A549 cells. However, higher expression of ZNF300 was detected in A549/DDP cells. Western blotting validated that ZNF300 expression was significantly higher in A549/DDP, H1650/DDP and H520/DDP cells compared to the corresponding progenitor cells. ZNF300 was endogenously overexpressed in H1915 and H446 cells. No significant difference in ZNF300 expression was observed between H1915/DDP and H1915 cells, or between H446/DDP and H446 cells (Figure 1A). The findings suggested that ZNF300 might mediate the chemoresistance of NSCLC rather than SCLC. BSP results (Figure 1B) and changes of ZNF300 expression in A549, H1650 and H520 cells after incubation of 5‐azacitidine or belinostat or combined usage of both (Figure 1C) demonstrated that ZNF300 expression was regulated by promoter methylation. In the following experiments, the recombinant lentivirus vectors were utilized to overexpress ZNF300 in A549 cells or silence ZNF300 in A549/DDP cells to explore the role of ZNF300 in the chemoresistance of NSCLC (Figure 1A).

As shown in Figure 1A, ZNF300 was overexpressed successfully in A549 cells by LV5‐ZNF300 (designated as A549‐ZNF300) and significantly silenced in A549/DDP cells by LV3‐shZNF300‐1661, LV3‐shZNF300‐2522, LV3‐shZNF300‐2659 and LV3‐shZNF300‐2777, respectively. Because LV3‐shZNF300‐2777 silenced ZNF300 most efficiently in A549/DDP cells, A549/DDP cells transfected with LV3‐shZNF300‐2777 (designated as A549/DDP‐shZNF300) were used in the subsequent experiments. A549 cells transfected with the lentivirus vector control LV5‐ZNF300‐NC (designated as A549‐ZNF300‐NC) and A549/DDP cells transfected with the lentivirus vector control LV3‐shZNF300‐NC (designated as A549/DDP‐shZNF300‐NC) were used as the corresponding controls. As displayed in Figure 1D, IC_50_ of cisplatin, gemcitabine, paclitaxel, docetaxel and pemetrexed on A549‐ZNF300 and A549/DDP‐shZNF300‐NC cells was significantly higher than that of A549‐ZNF300‐NC and A549/DDP‐shZNF300 cells. The apoptosis of A549‐ZNF300 and A549/DDP‐shZNF300‐NC cells induced by cisplatin was significantly lower than that of A549‐ZNF300‐NC and A549/DDP‐shZNF300 cells (Figure 1E), demonstrating that ZNF300 mediated the chemoresistance of NSCLC, which was confirmed in the subcutaneous tumour xenograft model. As shown in Figure 1F‐H, the cancerous tissues originating from the tumour xenograft models injected subcutaneously with A549‐ZNF300‐NC and A549/DDP‐shZNF300 cells presented a remarkable shrinkage in the tumour weight and volume when compared to that injected with A549‐ZNF300 and A549/DDP‐shZNF300‐NC cells after cisplatin was applied. The same results were obtained when A549/DDP cells were transfected with the LV3‐shZNF300‐1661 (data not shown), displaying that both LV3‐shZNF300‐2777 and LV3‐shZNF300‐1661 were specific for silencing ZNF300 in A549/DDP cells and no off‐target effects of LV3‐shZNF300 were observed in the present study.

### 3.2 ZNF300 promotes aggressive growth of tumour cells correlating to poor prognosis of patients with NSCLC

**FIGURE 2:**
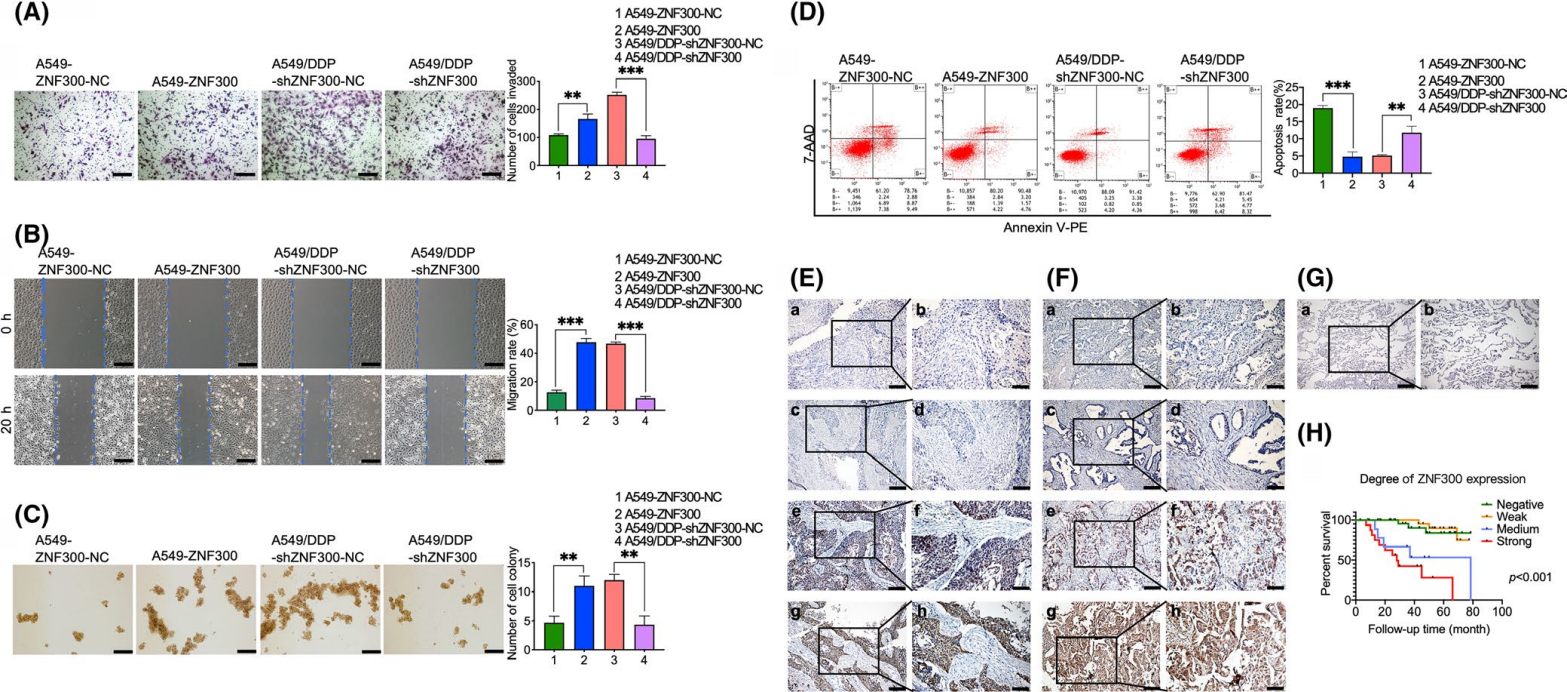
ZNF300 enhanced the aggressive growth of tumour cells and was correlated to the poor prognosis of NSCLC patients. Invasion (A), migration (B), colony forming (C) and anti‐anoikis apoptosis (D) of A549‐ZNF300‐NC, A549‐ZNF300, A549/DDP‐shZNF300‐NC and A549/DDP‐shZNF300 cells (a‐c: magnification: 100×, bar = 20 µm; data were expressed as the mean ± SD; significant difference between two groups: *P < .05, **P < .01, ***P < .001). Representative IHC staining of ZNF300 in lung squamous carcinoma (E), lung adenocarcinoma (F) and normal alveolar tissues (G) (Ea, Eb, Fa, Fb, Ga and Gb: negative ZNF300 staining; Ec, Ed, Fc and Fd: weak ZNF300 staining; Ee, Ef, Fe and Ff: medium ZNF300 staining; Eg, Eh, Fg and Fh: strong ZNF300 staining. Ea, Ec, Ee, Eg, Fa, Fc, Fe, Fg and Ga: magnification 100×, bar = 200 µm; Eb, Ed, Ef, Eh, Fb, Fd, Ff, Fh and Gb: magnification: 200×, bar = 100 µm). (H) Survival curve of lung adenocarcinoma patients with the stratified ZNF300 expression (OS data were from the commercial tissue microarray)

Clinically, once tumour cells acquire chemoresistance, the disease will progress malignantly. Hence, the biological characteristics were compared between chemoresistant cells and chemosensitive cells (A549‐ZNF300 vs A549‐ZNF300‐NC and A549/DDP‐shZNF300‐NC vs A549/DDP‐shZNF300). The transwell assay showed that the invasion of chemoresistant cells was significantly enhanced related to chemosensitive cells (number of cells invaded: 165.33 ± 14.70 vs 108.67 ± 3.40, *P* < .01, and 251.00 ± 7.87 vs 95.33 ± 8.99, *P* < .001, respectively, Figure 2A). The wounding healing assay presented that the migration of chemoresistant cells was significantly increased compared to chemosensitive cells (migration rate: 47.63 ± 2.14% vs 12.84 ± 1.03%, *P* < .001, and 46.64 ± 0.92% vs 8.64 ± 0.99%, *P* < .001, respectively, Figure 2B). And the anoikis resistance assay demonstrated that the colony forming (number of cell colony: 11.00 ± 1.41 vs 4.67 ± 0.94, *P* < .01, and 12.00 ± 0.82 vs 4.33 ± 1.25, *P* < .01, respectively, Figure 2C) and the anti‐anoikis apoptosis of chemoresistant cells (apoptosis rate: 4.79 ± 1.15% vs 18.97 ± 0.64%, *P* < .001, and 5.12 ± 0.21% vs 11.78 ± 1.57%, *P* < .01, respectively, Figure 2D) was strengthened compared to chemosensitive cells.

To evaluate the potential role of ZNF300 in lung cancer, we retrieved ZNF300 in normal human tissues (http://www.biogps.org/), lung cancer cell Lines (http://www.betastasis.com/) and lung cancer tissues (https://www.oncomine.org/). The retrieval revealed that no expression of ZNF300 was detected in the normal lung tissues (Figure S3). The median expression of ZNF300 in cell lines of NSCLC and SCLC was 121.68 and 266.32, respectively. The data of ZNF300 expression in cell lines of NSCLC and SCLC were downloaded from http://www.betastasis.com/tissues/cancer_cell_line_encyclopedia/gene_expression_barplot/, which were derived from the Cancer Cell Line Encyclopedia (CCLE) website (Affymetrix HG U133 Plus 2.0). According to the CCLE website, raw Affymetrix CEL files were converted to a single value for each probeset using RMA and quantile normalization. Based on the threshold of *P* < .05, FC > 2, gene rank: top 10%, data type: mRNA, no ZNF300 data of lung cancer were available in the datasets of cancer tissues vs normal tissues through an Oncomine Research for ZNF300 (Figure S4A). Interestingly, two ZNF300 datasets of lung cancer appeared in the outlier analysis (Broet‐Lung and Larsen‐Lung, Figure S4B,C). Because only age, sex and stage were listed in Broet‐Lung,^9^ we used the data from Larsen‐Lung to analyse the association of ZNF300 with the clinical characteristics of lung cancer patients.^10^ The analysis revealed that ZNF300 was associated with poor OS of NSCLC patients when ZNF300 expression was stratified into three levels of <0.00, 0‐0.25 and >0.25 (Figure S4D). Additionally, the expression of ZNF300 in anaplastic oligodendroglioma (Figure S4E,F), glioblastoma (Figure S4G), skin basal cell carcinoma (Figure S4H), invasive ductal breast carcinoma (Figure S4I) and invasive lobular breast carcinoma (Figure S4J) was significantly higher than that of the corresponding normal tissues. The analysis of biological characteristics and bioinformatics mentioned above strongly suggested that the upregulated ZNF300 induced by cisplatin might be of special importance in the invasion, metastasis and other malignant progression of NSCLC because SCLC, anaplastic oligodendroglioma, glioblastoma, skin basal cell carcinoma, invasive ductal breast carcinoma and invasive lobular breast carcinoma are all highly aggressive and malignant tumours.

ZNF300 staining on specimens from NSCLC patients and the tissue microarray by IHC validated that ZNF300 expression was positively correlated with stage, lymph node metastasis and relapse of NSCLC patients (Tables 1 and 2; Figure 2E‐H). Additionally, ZNF300 was displayed to be associated with the poor OS of patients with lung adenocarcinoma (Table 2; Figure 2H), confirming that ZNF300 mediated the malignancy of NSCLC.

**TABLE 1:** Correlation of ZNF300 with characteristics of patients with NSCLC (collected from Xinqiao Hospital)

Characteristic	ZNF300 expression					
	Case (n = 126) (%)	Negative (n = 64) (%)	Weak (n = 17) (%)	Medium (n = 38) (%)	Strong (n = 7) (%)	*P* value
Gender						
Male	88 (69.84)	40 (45.46)	13 (76.47)	30 (78.95)	5 (71.43)	.340^c^
Female	38 (30.16)	24 (63.16)	4 (23.53)	8 (21.05)	2 (28.57)	
Age at diagnosis (y)						
<60	77 (61.11)	37 (57.81)	11 (64.71)	25 (65.79)	4 (57.14)	.868^c^
≥60	49 (38.89)	27 (42.19)	6 (35.29)	13 (34.21)	3 (42.86)	
Histology						
Adenocarcinoma	60 (47.62)	29 (45.31)	8 (47.06)	21 (55.26)	2 (28.57)	.782^c^
Squamous carcinoma	55 (43.65)	29 (45.31)	7 (41.18)	14 (36.84)	5 (71.43)	
Adenosquamous carcinoma	11 (8.73)	6 (4.76)	2 (11.76)	3 (7.90)	0 (0.00)	
Pack‐years of smoking^a^						
<30	91 (72.22)	47 (73.44)	13 (76.47)	25 (65.79)	6 (85.71)	.715^c^
≥30	35 (27.78)	17 (26.56)	4 (23.53)	13 (34.21)	1 (14.29)	
Pathological grade						
Well	57 (45.24)	31 (48.44)	7 (41.18)	14 (36.84)	5 (71.43)	.248^d^
Moderately	58 (46.03)	25 (39.06)	9 (52.94	22 (57.90)	2 (28.57)	
Poorly	11 (8.73)	8 (12.50)	1 (5.88)	2 (5.26)	0 (0.00)	
Clinical stage (TNM)						
I	83 (65.87)	52 (81.25)	6 (35.29)	25 (65.79)	0 (0.00)	.002^d^
II	17 (13.49)	6 (9.38)	6 (35.29)	5 (13.16)	0 (0.00)	
III	23 (18.25)	6 (9.38)	5 (29.41)	7 (18.42)	5 (71.43)	
IV	3 (2.38)	0 (0.00)	0 (0.00)	1 (2.63)	2 (28.57)	
Lymphatic invasion (pre‐surgery)						
(+)	34 (26.98)	4 (6.25)	8 (47.06)	17 (44.74)	5 (71.43)	.007^d^
(−)	92 (73.02)	60 (93.75)	9 (52.94)	21 (55.26)	2 (28.57)	
Lymphatic invasion (post‐surgery)^b^						
(+)	16 (12.70)	5 (7.81)	1 (5.88)	6 (15.79)	4 (57.14)	.005^d^
(−)	90 (71.43)	50 (81.25)	10 (58.82)	28 (73.68)	2 (28.57)	
No date	20 (15.87)	9 (10.94)	6 (35.30)	4 (10.53)	1 (14.29)	
Regional metastasis						
(+)	22 (17.46)	11 (17.19)	2 (11.76)	5 (13.18)	4 (57.14)	.325^d^
(−)	83 (65.87)	45 (70.31)	10 (58.82)	26 (68.42)	2 (28.57)	
No data	21 (16.67)	8 (12.50)	5 (29.41)	7 (18.42)	1 (14.29)	
Relapse						
(+)	14 (11.11)	5 (7.81)	2 (11.76)	4 (10.53)	3 (42.86)	.036^d^
(−)	90 (71.43)	51 (79.69)	9 (52.94)	28 (73.68)	2 (28.57)	
No data	22 (17.16)	8 (12.50)	6 (35.29)	6 (15.79)	2 (28.57)	
Changes of tumour size						
No changes of residual tumour	24 (19.05)	13 (20.31)	3 (17.65)	7 (18.42)	1 (14.29)	.107^d^
Tumour increased	12 (9.52)	4 (6.25)	2 (11.76)	5 (13.16)	1 (14.29)	
Tumour regressed	8 (6.35)	5 (7.81)	1 (5.88)	2 (5.26)	0 (0.00)	
No cancer cells after surgery	61 (48.41)	34 (26.98)	6 (35.29)	17 (44.74)	4 (57.14)	
No data	21 (16.67)	8 (6.35)	5 (29.41)	7 (18.42)	1 (14.29)	

^a^ Median number of pack‐years was used as the cut‐off point.

^b^ Patients who received adjuvant cisplatin‐ or carboplatin‐based chemotherapy after surgery.

^c^ Chi‐square test or Fisher's exact test.

^d^ Adjusted *P* value determined by multivariate logistic regression analysis, adjusted for gender, age and pack‐years of cigarette smoking.

**TABLE 2:** Correlation of ZNF300 with clinical characteristics of patients with lung adenocarcinoma (tissue microarray)

Characteristics	ZNF300 expression					
	Cases (n = 80)	Negative (n = 30) (%)	Weak (n = 25) (%)	Medium (n = 9) (%)	Strong (n = 16) (%)	*P* value
Gender						
Male	30 (37.50)	11 (36.67)	8 (32.00)	4 (44.44)	7 (43.75)	.852^b^
Female	50 (62.50)	19 (63.33)	17 (68.00)	5 (55.56)	9 (56.25)	
Age at diagnosis (y)						
<60	43 (53.75)	20 (66.67)	10 (40.00)	4 (44.44)	9 (56.25)	.233^b^
≥60	37 (46.25)	10 (33.33)	15 (60.00)	5 (55.56)	7 (43.75)	
Pack‐years of smoking^a^						
<30	73 (91.25)	28 (93.33)	24 (100.00)	6 (88.89)	15 (100.00)	.088^b^
≥30	7 (8.75)	2 (6.67)	1 (0.00)	3 (11.11)	1 (0.00)	
Pathological grade						
Well	26 (32.50)	13 (43.33)	11 (44.00)	0 (0.00)	2 (12.50)	.006^c^
Moderately	31 (38.75)	7 (23.33)	13 (52.00)	8 (88.89)	3 (18.75)	
Poorly	23 (28.75)	10 (33.33)	1 (4.00)	1 (11.11)	11 (68.75)	
Clinical stage (TNM)						
I	42 (52.50)	17 (56.67)	21 (84.00)	3 (33.33)	1 (6.25)	.002^c^
II	10 (12.50)	3 (10.00)	3 (12.00)	0 (0.00)	4 (25.00)	
III	19 (23.75)	5 (16.67)	1 (4.00)	6 (66.67)	7 (43.75)	
IV	9 (11.25)	5 (16.67)	0 (0.00)	0 (0.00)	4 (25.00)	
Lymphatic invasion (pre‐surgery)						
(+)	27 (33.75)	6 (20.00)	3 (12.00)	5 (55.56)	13 (81.25)	.001^c^
(−)	53 (66.25)	24 (80.00)	22 (88.00)	4 (44.44)	3 (18.75)	
Regional metastasis						
(+)	14 (17.50)	5 (16.67)	0 (0.00)	1 (11.11.)	8 (50.00)	.015^c^
(−)	66 (82.50)	25 (83.33)	25 (100.00)	8 (88.89.)	8 (50.00)	
Relapse						
(+)	39 (48.75)	14 (46.67)	13 (52.00)	7 (77.78)	5 (31.25)	.013^c^
(−)	41 (51.25)	16 (53.33)	12 (48.00)	2 (22.22)	11 (68.75)	
OS (mo)						
<36	30 (37.50)	11 (36.67)	4 (16.00)	4 (44.44)	11 (68.75)	.017^c^
36‐60	28 (35.00)	10 (33.33)	10 (40.00)	4 (44.44)	4 (25.00)	
>60	22 (27.50)	9 (30.00)	11 (44.00)	1 (11.11)	1 (6.25)	

^a^ Median number of pack‐years was used as the cut‐off point.

^b^ Chi‐square test or Fisher's exact test.

^c^ Adjusted *P* value determined by multivariate logistic regression analysis, adjusted for gender, age and pack‐years of cigarette smoking.

### 3.3 ZNF300 inhibits MAPK/ERK signalling pathway and activates CDK1 by inhibiting WEE1 and MYT1 and modulating MYC/AURKA/BORA/PLK1 axis

**FIGURE 3:**
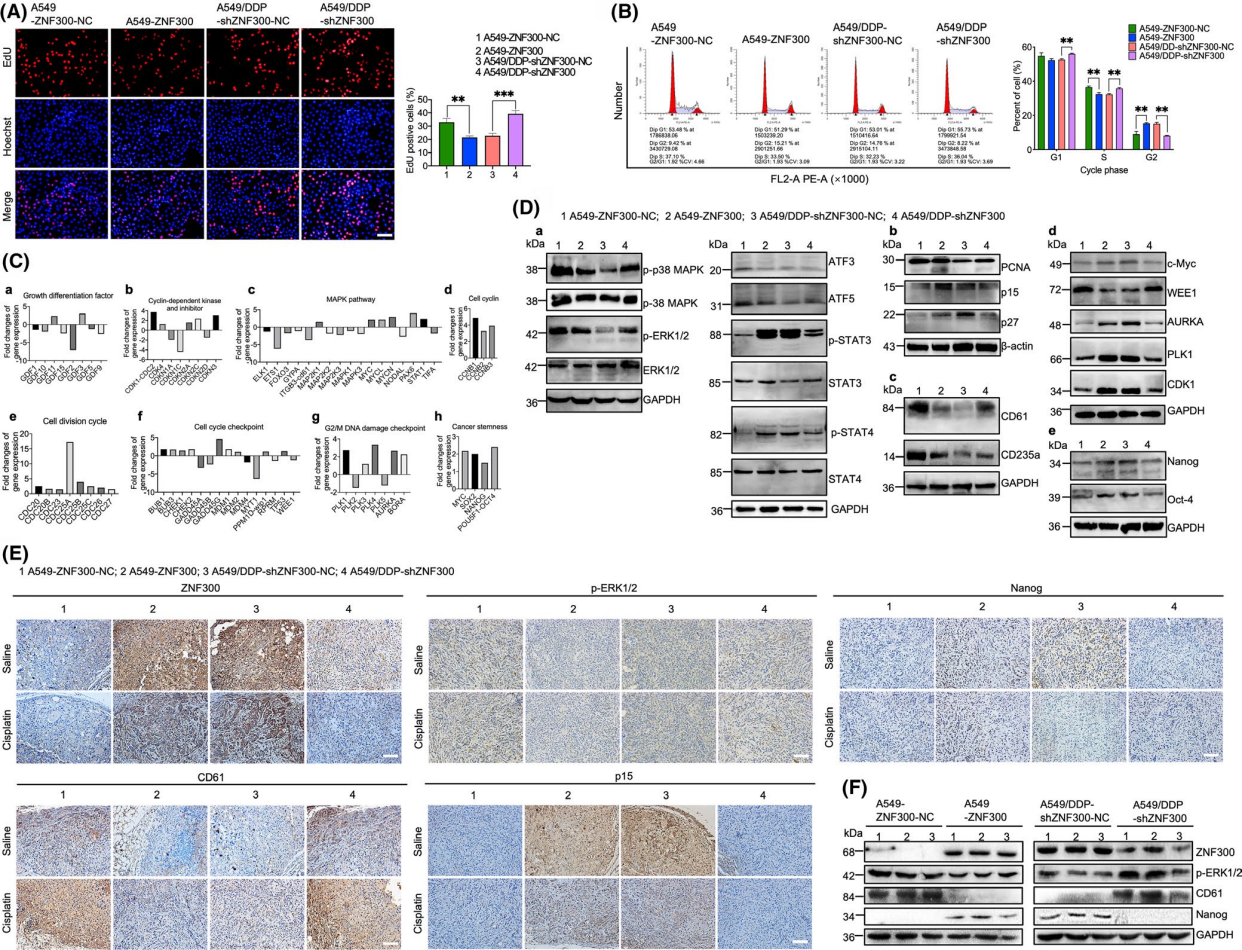
ZNF300 inhibited MAPK/ERK pathways and activated CDK1 by inhibiting WEE1 and MYT1 and modulating MYC/AURKA/BORA/PLK1 axis. Proliferation (A) (magnification: 100×, bar = 200 µm) and cell cycle (B) of A549‐ZNF300‐NC, A549‐ZNF300, A549/DDP‐shZNF300‐NC and A549/DDP‐shZNF300 cells. Fold changes (FC) of the differentially expressed genes related with proliferation, growth, differentiation and cell cycle between A549‐ZNF300‐NC and A549‐ZNF300 cells (Ca‐Ch). Validation of the differentially expressed core genes between A549‐ZNF300‐NC and A549‐ZNF300 cells using Western blotting (Da‐De). IHC staining (E) (magnification: 200×, bar = 100 µm) and Western blotting analysis (F) of ZNF300, p‐ERK1/2, Nanog, CD61 and p15 in the tumour tissues of xenograft models

ZNF300 was reported to function as a transcriptional regulator; hence, we compared the expression profiles between A549‐ZNF300‐NC and A549‐ZNF300 cells using Affymetrix Human U133 Plus 2.0. The microarray data have been uploaded to the GEO database (https://www.ncbi.nlm.nih.gov/geo/) with the accession number GSE145880. Because the cells with upregulated ZNF300 (A549‐ZNF300 and A549/DDP‐shZNF300‐NC) manifested relatively slower proliferation (Figure 3A) and cell cycle arrest at G2 phase compared to the cells with downregulated ZNF300 (A549‐ZNF300‐NC and A549/DDP‐shZNF300) (Figure 3B), we screened the genes that might regulate these biological characteristics based on the keywords of proliferation, growth, differentiation and cell cycle in the columns of gene.title or pathway of the expression profiles (A549‐ZNF300 vs A549‐ZNF300‐NC). MAPK was also screened as a keyword in the column of pathway because of its important function in growth and differentiation of cells. The screening displayed that most of the genes linked with MAPK/ERK pathways were downregulated, while most of the genes associated with cell cycle and cancer stemness were upregulated in A549‐ZNF300 cells compared to A549‐ZNF300‐NC cells. The related genes are summarized in Table S2 and analysed by gene set enrichment analysis (GSEA), which showed the enrichment plots (Figure S5A). According to their functions in cell cycle, we classified the cell cycle–related genes into kinase inhibitor, kinase, cell cycle checkpoint, cell division cycle, cell cyclin and G2/M DNA damage checkpoint, and found that most of the genes related with cyclin‐dependent kinase inhibitor were downregulated, while most of the genes related with cyclin‐dependent kinase, cell cycle checkpoint, cell division cycle, cell cyclin and G2/M DNA damage checkpoint were upregulated in A549‐ZNF300 compared to A549‐ZNF300‐NC (Figure 3C). The core genes are shown in Figure 3Ca‐h, verified by the Western blotting of the key genes (including PCNA, p15, p27, CD61, CD235a, p38 MAPK, ERK1/2, ATF3, ATF5, STAT3, STAT4, MYC, WEE1, AURKA, PLK1, CDK1, Nanog and Oct‐4, Figure 3Da‐e).

Moreover, the Western blotting and IHC staining of the key genes on the tumour tissues from subcutaneous xenograft further proved that p‐p38 MAPK, p‐ERK1/2 (encoded by genes *MAPK14* and *MAPK1* */MAPK3*, respectively) and CD61 were inhibited, while p15 and Nanog were upregulated in the tumour tissues of xenograft models injected with A549‐ZNF300 and A549/DDP‐shZNF300‐NC cells compared to that injected with A549‐ZNF300‐NC and A549/DDP‐shZNF300 cells (Figure 3E,F).

**FIGURE 4:**
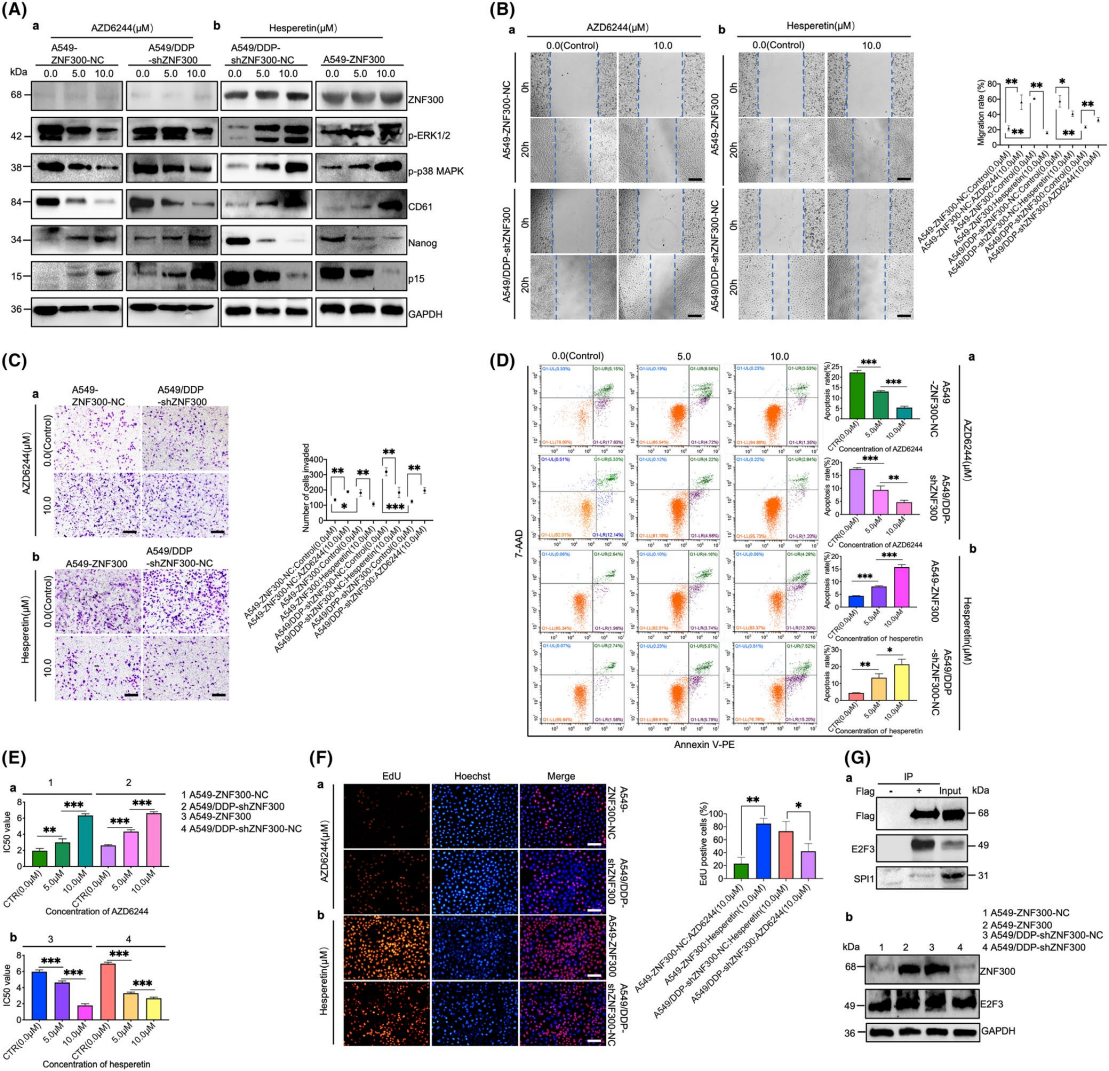
ZNF300 was the upstream regulator of MAPK/ERK pathways. Western blotting analysis of ZNF300, p‐ERK1/2, p‐p38 MAPK, CD61, Nanog and p15 in A549‐ZNF300‐NC and A549/DDP‐shZNF300 cells after treatment with AZD6244 (Aa), and in A549/DDP‐shZNF300‐NC and A549‐ZNF300 cells after treatment with hesperetin (Ab). Migration of A549‐ZNF300‐NC and A549/DDP‐shZNF300 cells after treatment with AZD6244 (Ba), and of A549‐ZNF300 and A549/DDP‐shZNF300‐NC cells after treatment with hesperetin (Bb) compared to the controls (magnification: 100×, bar = 200 µm). Invasion of A549‐ZNF300‐NC and A549/DDP‐shZNF300 cells after treatment with AZD6244 (Ca), and of A549‐ZNF300 and A549/DDP‐shZNF300‐NC cells after treatment with hesperetin (Cb) compared to the controls (magnification: 100×, bar = 200 µm). Apoptosis of A549‐ZNF300‐NC and A549/DDP‐shZNF300 cells incubated with AZD6244 (Da), and of A549‐ZNF300 and A549/DDP‐shZNF300‐NC cells incubated with hesperetin (Db), followed by 2.0 µg/mL cisplatin treatment. IC_50_ of cisplatin on A549‐ZNF300‐NC and A549/DDP‐shZNF300 cells after application of AZD6244 (Ea), and on A549‐ZNF300 and A549/DDP‐shZNF300‐NC cells after application of hesperetin (Eb). The proliferation of A549‐ZNF300‐NC and A549/DDP‐shZNF300 cells after treatment with AZD6244 (Fa), and A549‐ZNF300 and A549/DDP‐shZNF300‐NC cells after treatment with hesperetin (Fb) compared to the controls using EdU staining (magnification: 100×, bar = 200 µm). Co‐IP assay of ZNF300 interacting with E2F3 and SPI1 (Ga) and Western blotting analysis of ZNF300 and E2F3 in A549‐ZNF300‐NC, A549‐ZNF300, A549/DDP‐shZNF300‐NC and A549/DDP‐shZNF300 cells (Gb). All the data were expressed as the mean ± SD (significant difference between two groups: *P < .05, **P < .01, ***P < .001)

To clarify the relationship between ZNF300 and MAPK/ERK pathways, AZD6244, an inhibitor, and hesperetin, an agonist of MAPK/ERK pathways, were used to treat the relevant cells. As displayed in Figure 4Aa, p‐ERK1/2, p‐p38 MAPK, and CD61 decreased, while p15 and Nanog increased in A549‐ZNF300‐NC and A549/DDP‐shZNF300 cells after treatment with AZD6244. Additionally, the migration (Figure 4Ba), invasion (Figure 4Ca), anti‐apoptosis (Figure 4Da) and resistance to cisplatin (Figure 4Ea) of A549‐ZNF300‐NC and A549/DDP‐shZNF300 cells were significantly enhanced, but proliferation was subdued after treatment with AZD6244 (Figure 4Fa). In contrast, hesperetin could reverse the above results (Figure 4Ab,Bb,Cb,Db,Fb). No effect of AZD6244 and hesperetin was observed on the ZNF300 expression (Figure 4Aa,b), strongly indicated that ZNF300 was the upstream regulator of MAPK/ERK pathways.

ZNF300 was predicted to interact with E2F3 and SPI1 (https://www.gcbi.com.cn/). The immunoblotting showed that ZNF300‐flag, used as bait, could bind to E2F3 (Figure 4Ga), indicating that ZNF300 might interact with E2F3 to function in the chemoresistance and aggressive behaviour of tumour cells. As shown in Figure 4Gb, there was no significant difference of E2F3 expression between the cells with upregulated ZNF300 and the cells with downregulated ZNF300, implying that E2F3 might not be regulated by ZNF300.

### 3.4 ICA and ATRA improve the anti‐tumour effect of cisplatin on chemoresistant cells by inducing differentiation

**FIGURE 5:**
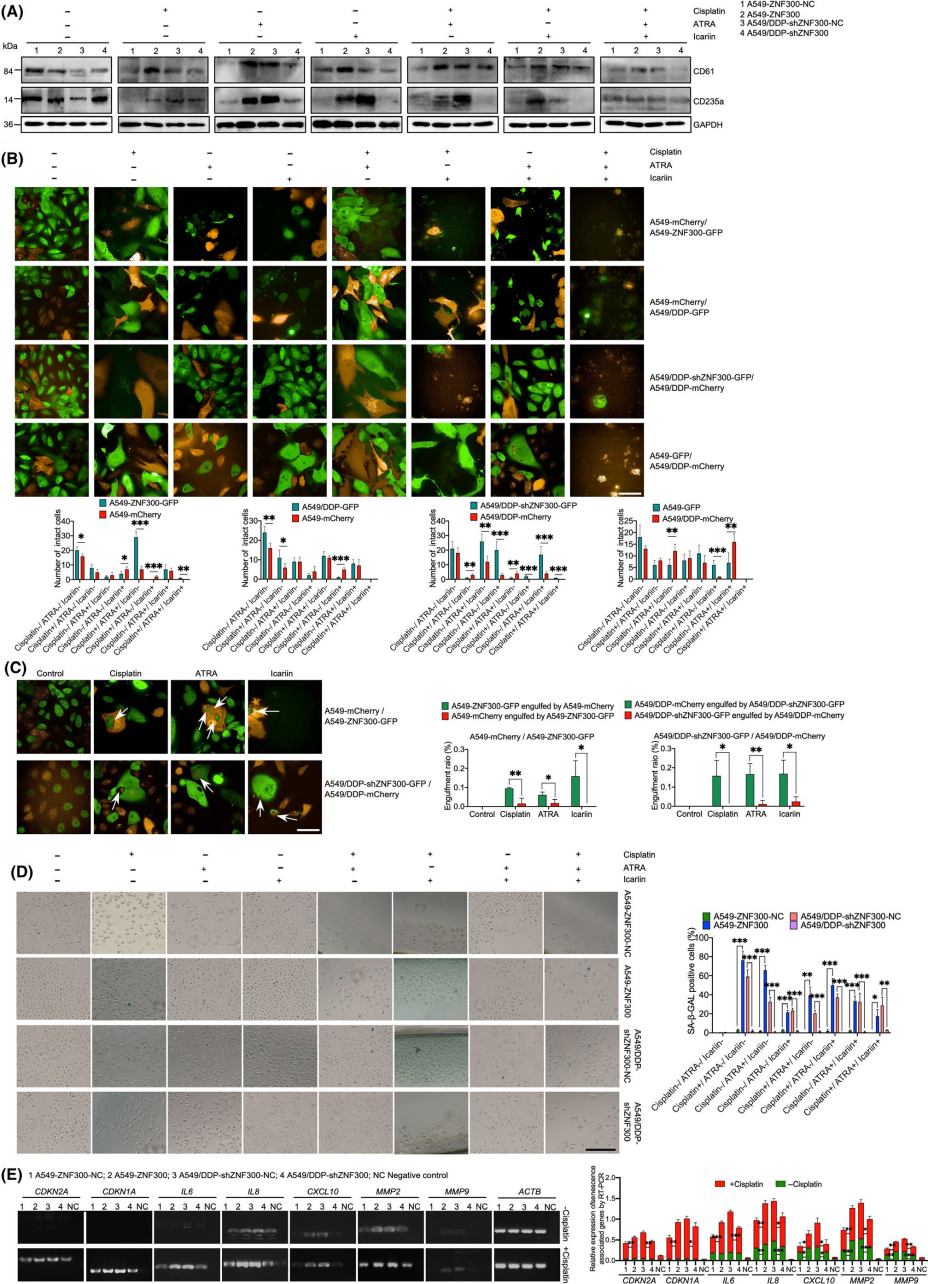
ICA and ATRA improved the anti‐tumour effect of cisplatin on the chemoresistant cells by inducing differentiation, and the chemosensitive cell engulfed the chemoresistant cells at a higher proportion to obtain a survival advantage under the pressure caused by chemotherapeutic drugs. A, Expression of CD61 and CD235a in A549‐ZNF300‐NC, A549‐ZNF300, A549/DDP‐shZNF300‐NC and A549/DDP‐shZNF300 cells after treatment with cisplatin, ATRA and ICA, separately or combinedly. B, Images of the co‐cultured cells at the treatment endpoint of cisplatin, ATRA, ICA, separately or combinedly, for 7 d (magnification: 630×, bar = 50 µm). C, Representative engulfment images of the co‐cultured cells after treatment with cisplatin, ATRA and ICA for 72 h (magnification: 630×, bar = 50 µm). D, SA‐β‐GAL staining of the senescent cells (magnification: 200×, bar = 100 µm). E, Expression of genes related with senescence and SASP determined by RT‐PCR in the four cells with or without the treatment of 2 µg/mL cisplatin for 48 h. All the data were expressed as the mean ± SD (significant difference between two groups: *P < .05, **P < .01, ***P < .001)

All‐trans retinoic acid (ATRA) and icariin (ICA) were reported to induce the cell differentiation.^11^, ^12^ Thus, we used the two chemical compounds to test whether they could enhance the anti‐tumour effect of cisplatin on the chemoresistant cells by inducing differentiation. As shown in Figure 5A, compared to the chemosensitive cells (A549‐ZNF300‐NC and A549/DDP‐shZNF300), CD235a and CD61 upregulated in the chemoresistant cells (A549‐ZNF300 and A549/DDP‐shZNF300‐NC) after treatment with ICA and ATRA, respectively. Observation of the co‐cultured cells for 7 days demonstrated that the chemoresistant cells (A549‐ZNF300‐GFP, A549/DDP‐mCherry and A549/DDP‐shZNF300‐NC‐GFP) were more sensitive to cisplatin combined with ATRA or ICA compared to the chemosensitive cells (A549‐mCherry, A549‐ZNF300‐NC‐GFP and A549/DDP‐shZNF300‐GFP), suggesting that ATRA and ICA could enhance the anti‐tumour effect of cisplatin on the chemoresistant cells by inducing the differentiation of tumour cells. Additionally, the synergistic cytotoxic effect of cisplatin with ICA on the chemoresistant cells was stronger than that of the combination of cisplatin and ATRA (Figure 5B). The videos will be provided upon request.

Intriguingly, the treatment with cisplatin, ATRA and ICA, separately or combined, led to a remarkable mutual phagocytosis response in both the chemoresistant cells and chemosensitive cells. It is noteworthy here that the ratio of the chemoresistant cells engulfed by the chemosensitive cells was relatively higher than that of the chemosensitive cells engulfed by the chemoresistant cells (Figure 5C). The senescence of tumour cells induced by chemotherapy was reported to enhance their survival via engulfing both neighbouring senescent or non‐senescent tumour cells.^13^ Thus, we utilized SA‐β‐GAL staining to determine whether the mutual engulfment of the chemosensitive cells and the chemoresistant cells was related with the senescence induced by chemotherapeutic agents. The staining manifested that the positively stained chemoresistant cells of SA‐β‐GAL were more abundant than that of the chemosensitive cells after treated by cisplatin combined with ATRA and ICA, especially ICA (Figure 5D). The expression of genes related with senescence (cyclin‐dependent kinase inhibitor 2A (*CDKN2A*) and cyclin‐dependent kinase inhibitor 1 (*CDKN1A*)) and senescence‐associated secreting phenotype (SASP) (interleukin‐6 (*IL6*), interleukin‐8 (*IL8*), C‐X‐C motif chemokine ligand 10 (*CXCL10*), matrix metalloproteinase‐2 (*MMP2*) and matrix metalloproteinase‐9 (*MMP9*)) at the mRNA level was determined by RT‐PCR. As shown in Figure 5E, the expression of *CDKN2A*, *CDKN1A*, *IL6*, *IL8*, *CXCL10*, *MMP2* and *MMP9* significantly increased in chemoresistant cells after treatment with cisplatin compared to chemosensitive cells, confirming that cisplatin could induce more chemoresistant cells into senescent stage compared to chemosensitive cells.

## 4 DISCUSSION

Chemoresistance induced by cisplatin has become the major impediment to lung cancer chemotherapy, resulting in the failure of drug combination regimen in lung cancer. Therefore, it is important to continue the exploration of chemoresistance mechanisms in NSCLC. In the study, an increased expression of ZNF300 was detected in three chemoresistant cell lines of NSCLC. The bioinformatic analysis and ZNF300 staining on the specimens of patients with NSCLC showed that ZNF300 was associated with the poor OS of patients with NSCLC. Silencing ZNF300 in A549/DDP cells (A549/DDP‐shZNF300) and overexpressing ZNF300 in A549 cells (A549‐ZNF300) demonstrated that the cells with upregulated ZNF300 (A549‐ZNF300 and A549/DDP‐shZNF300‐NC) exhibited resistance to cisplatin, gemcitabine, paclitaxel, docetaxel and pemetrexed, enhanced invasion and metastasis, a reduced fraction of anoikis cells, and decreased apoptosis induced by cisplatin compared to the cells with downregulated ZNF300 (A549‐ZNF300‐NC and A549/DDP‐shZNF300). Thereby, confirming that the cisplatin‐induced upregulation of ZNF300 promoted the chemoresistance and aggressive growth of NSCLC. Further, this provided evidence that the aggressive growth of tumour cells was enhanced constantly with the development of chemoresistance.^14^ The experimental results in vitro were validated in the subcutaneous xenografts of nude mice in vivo.

*ZNF300* gene was originally isolated from a human embryo and overexpressed in the heart, brain, skeletal muscle and testis.^15^, ^16^ Being a typical member of the zinc finger protein family, ZNF300 was reported to exert its transcriptional impact in the nucleus via the KRAB domain.^15^ It has been shown to play a pivotal role in cell differentiation,^17^ stimulation of fatty acid oxidation and alleviation of hepatosteatosis by regulating PPARα,^18^ and regulation of IL‐2Rβ promoter activity by binding directly to the overlapping site of ZNF300/EGR1.^19^ Additionally, ZNF300 was reported to promote the growth and metastasis of HeLa cells by regulating the NF‐kB pathway via interacting with IKKβ and activating TRAF2 and enhance the malignant progression of leukaemia after being activated by PU.1.^20^, ^21^ Since ZNF300 was one of the three methylated genes identified in the plasma cell‐free DNA (cfDNA) of hepatocellular carcinoma as compared to liver tissue DNA, it can be used as a biomarker for early detection and high‐risk screening of hepatocellular carcinoma.^22^ ZNF300 expression was higher in the desmoid tumours than in the normal tissues, but its function is still unknown.^23^

Since the cells with upregulated ZNF300 presented slower proliferation and slower cell cycling compared to the cells with downregulated ZNF300, we focused on the differentially expressed genes related with proliferation, growth, differentiation and cell cycle between A549‐ZNF300‐NC and A549‐ZNF300 cells. The analysis displayed that most of the genes associated with growth, differentiation, MAPK/ERK pathways and cyclin‐dependent kinase inhibitor were downregulated, whereas most of the genes associated with cyclin‐dependent kinase, cell cycle checkpoint, cell division, cell cyclins, G2/M DNA damage checkpoint and cancer stemness were upregulated in A549‐ZNF300 cells compared to A549‐ZNF300‐NC cells.

MAPK/ERK pathways play important roles in regulating cell growth, development, division, death, proliferation, differentiation and various other physiological processes.^24^ Cyclin‐dependent kinase 1 (CDK1/cdc2) is essential for cell cycle progression and cell division. During the period of G2/M transformation regulated by CDK1‐cyclin B complex, CDK1 is inactivated by the tyrosine kinases WEE1 and MYT1.^25^ Meanwhile, when the cell enters the M phase, the kinase Aurora A (AURKA) and its cofactor BORA synergistically activate PLK1, which in turn activates the activity of phosphatase CDC25 and downstream CDK1 to drive the cell into mitosis by establishing an effective feedback loop.^26^ Inhibition of CDK1 expression in the normal cells increased the sensitivity of cells to DNA damage.^27^ Being an important regulator of cell cycle progression and mitosis, AURKA has been described as an oncoprotein and a therapeutic target in various types of advanced tumours. Additionally, MYC was shown to enable the tumour cells to overcome a latent cell cycle arrest in G2/M phase by directly binding AURKA. Inhibition of the MYC‐AURKA interaction by conformation‐changing AURKA inhibitors resulted in the subsequent MYC degradation and cell death.^28^ Thus, it was deduced that the upregulated ZNF300 induced by cisplatin might inhibit MAPK/ERK pathway to suppress the proliferation and differentiation of tumour cells to promote them to enter a relatively slower growth or even a quiescent or senescent state to avoid the cytotoxic effect of chemotherapeutic drugs. Meanwhile, ZNF300 might activate CDK1 through inhibition of WEE1 and MYT1 and modulation of the MYC/AURKA/BORA/PLK1 axis to promote the tumour cells to overcome G2 arrest and enter the M phase to avoid the apoptosis induced by chemotherapeutic drugs. Once the survival pressure caused by chemotherapeutic drugs disappeared or was removed (periodicity of chemotherapeutic management), the surviving tumour cells acquired chemoresistance and grew aggressively to promote the malignant progression of diseases.

Our findings that the chemoresistance and malignant progression of NSCLC were caused by the slow‐cycling population was consistent with the prior studies. In the previous studies, the slow‐cycling cells were shown to drive a highly invasive cell state that leads to increased tumour cell dissemination, metastasis and invasion compared to cycling cells in melanoma.^29^, ^30^ Recently, the wild‐type p53 expression has been revealed to be a driver of therapy resistance by initiating a slow‐cycling phenotype in melanoma.^31^ In the study, we also observed a slow‐cycling phenotype and higher expression of p53 in chemoresistant cells compared to chemosensitive cells, but there was no significant difference of p53 expression between A549‐ZNF300 and A549‐ZNF300‐NC cells, or between A549/DDP‐shZNF300 and A549/DDP‐shZNF300‐NC cells (Figure S5B), demonstrating that p53 might be involved in the chemoresistance and malignant progression of NSCLC though not be regulated by ZNF300. Additionally, the retrieval of p53 status in the ‘Handbook of p53 mutation in cell lines’ (http://p53.free.fr/Database/Cancer_cell_lines/cell_lines_1.0.pdf) and p53 expression in the betastasis (http://www.betastasis.com) showed that p53 is wild type in cell lines of A549, H1650 and H446, while mutant in cell lines of H1915 and H520. Relatively, the expression of p53 is higher in A549 and H446 cells, while weak in H1650, H1915 and H520 cells (Figure S5C). The role of p53 in the chemoresistance and malignant progression of NSCLC will be explored in our future work.

Because many drugs, including DNA damaging drugs, induce senescence and SA‐β‐GAL positivity in A549 cells, one of the explanations for the phenomena that A549 cells with downregulated ZNF300 failed to turn positive in the study might be that a 48‐hour time point is early for SA‐β‐GAL detection. Additionally, though the morphologies of some A549 cells with downregulated ZNF300 presented a senescent phenotype after treated by the drugs, these cells were hardly stained by SA‐β‐GAL or SA‐β‐GAL staining was very weak, resulting in the lesser number of SA‐β‐GAL positively stained cells. Furthermore, 2 µg/mL cisplatin, 5 µg/mL ATRA and 20 µg/mL ICA, the IC_50_ concentration of the three drugs on A549 cells, were lower dose to A549 cells with upregulated ZNF300 (chemoresistant cells) related to A549 cells with downregulated ZNF300 (chemosensitive cells). Thus, we deduced that the concentrations of the three drugs used for senescence detection in the study could induce the apoptosis of chemosensitive cells but incur the senescence of chemoresistant cells, which was consistent with the prior studies that the lower doses of resveratrol treatment inhibited lung cancer cell growth by the induction of premature senescence through ROS‐mediated DNA damage in a dose‐dependent manner, while higher doses may induce apoptosis in tumour cells.^32^

Several limitations exist in this study; firstly, since the collection of tumour specimens from patients who present the chemoresistant phenotype is difficult, the current research could not provide the clinical evidence of the ZNF300 association with cisplatin‐induced chemoresistance. Secondly, the nude mice were not treated with ATRA or ICA; therefore, the effects of the two chemicals on the tumour growth in vivo could not be observed. Nevertheless, the research provided a novel insight into the development of chemoresistance and a potential tactic to combat it.

Taken together, the findings of the study suggest that ZNF300 might mediate the chemoresistance and malignant progression of NSCLC. The probable mechanism of ZNF300 could be through suppressing cell proliferation and differentiation by downregulating MAPK/ERK pathways and slowing cell cycle via activating CDK1 by inhibiting WEE1/MYT1 and modulating MYC/AURKA/BORA/PLK1 axis. Cisplatin, combined with ATRA and ICA, especially ICA, might be beneficial for the chemoresistant cases of NSCLC.

## CONFLICT OF INTEREST

The authors declare no conflict of interest.

## AUTHOR CONTRIBUTIONS

Shilong Yu, Zhi Ao and Xianhui Wang performed the experiments in vitro. Shilong Yu, Yi Wu and Liyuan Song designed and conducted the animal experiments. Shilong Yu, Peng Zhang and Xiaokang Li, Min Liu, Xihua Li, and Xuanbin Wang participated in the data analysis. Ruijie Zhang, Pin Qian and Yan Chen collected clinical data and prepared paraffin section. Fuyun Ji and Guisheng Qian conceived the study. Fuyun Ji, Guisheng Qian and Xuzhi Ruan participated in the study design. Shilong Yu and Fuyun Ji wrote the manuscript. Guisheng Qian and Xuzhi Ruan revised the manuscript. All authors critically reviewed the manuscript and approved the final version for publication.

## ETHICS APPROVAL AND CONSENT

Informed consent was obtained from all the patients participating in this study. All animal experiments were approved by the Experimental Animal Ethics Committee of Xinqiao Hospital of the Army Medical University [SYXK(YU)2012‐0011] and Hubei University of Medicine [SYXK(E)2016‐0031].

## Supporting information

Fig S1

Fig S2

Fig S3

Fig S4

Fig S5

Table S1

Table S2

Doc S1

## Data Availability

The microarray data have been uploaded to the GEO database (https://www.ncbi.nlm.nih.gov/geo/) with the accession number GSE145880 for A549‐ZNF300 cells vs A549‐ZNF300‐NC cells and GSE154243 for A549/DDP cells vs A549 cells.

## References

[1] Bray F , Ferlay J , Soerjomataram I , et al. Global cancer statistics 2018: GLOBOCAN estimates of incidence and mortality worldwide for 36 cancers in 185 countries. CA Cancer J Clin. 2018;68(6):394‐424. 30207593 10.3322/caac.21492

[2] Hirsch FR , Suda K , Wiens J , et al. New and emerging targeted treatments in advanced non‐small‐cell lung cancer. Lancet. 2016;388(10048):1012‐1024. 27598681 10.1016/S0140-6736(16)31473-8

[3] Forde PM , Chaft JE , Smith KN , et al. Neoadjuvant PD‐1 blockade in resectable lung cancer. N Engl J Med. 2018;378(21):1976‐1986. 29658848 10.1056/NEJMoa1716078 PMC6223617

[4] Galluzzi L , Senovilla L , Vitale I , et al. Molecular mechanisms of cisplatin resistance. Oncogene. 2012;31(15):1869‐1883. 21892204 10.1038/onc.2011.384

[5] Waghray D , Zhang Q . Inhibit or evade multidrug resistance P‐glycoprotein in cancer treatment. J Med Chem. 2018;61(12):5108‐5121. 29251920 10.1021/acs.jmedchem.7b01457 PMC6281405

[6] Guo R , Wu G , Li H , et al. Promoter methylation profiles between human lung adenocarcinoma multidrug resistant A549/cisplatin (A549/DDP) cells and its progenitor A549 cells. Biol Pharm Bull. 2013;36(8):1310‐1316. 23902976 10.1248/bpb.b13-00153

[7] Zhang R , Yao W , Qian P , et al. Increased sensitivity of human lung adenocarcinoma cells to cisplatin associated with downregulated contactin‐1. Biomed Pharmacother. 2015;71(7):172‐184. 25960233 10.1016/j.biopha.2014.11.004

[8] Ao Z , Yu S , Qian P , et al. Tumor angiogenesis of SCLC inhibited by decreased expression of FMOD via downregulating angiogenic factors of endothelial cells. Biomed Pharmacother. 2017;87:539‐547. 28081464 10.1016/j.biopha.2016.12.110

[9] Broet P , Camilleri‐Broet S , Zhang S , et al. Prediction of clinical outcome in multiple lung cancer cohorts by integrative genomics: implications for chemotherapy selection. Cancer Res. 2009;69(3):1055‐1062. 19176396 10.1158/0008-5472.CAN-08-1116

[10] Larsen JE , Pavey SJ , Passmore LH , et al. Expression profiling defines a recurrence signature in lung squamous cell carcinoma. Carcinogenesis. 2007;28(3):760‐766. 17082175 10.1093/carcin/bgl207

[11] Martino OD , Welch JS . Retinoic acid receptors in acute myeloid leukemia therapy. Cancers. 2009;11(12):pii1915. 10.3390/cancers11121915 PMC6966485 31805753

[12] Tang Y , Jacobi A , Vater C , et al. Icariin promotes angiogenic differentiation and prevents oxidative stress‐induced autophagy in endothelial progenitor cells. Stem Cells. 2015;33:1863‐1877. 25787271 10.1002/stem.2005

[13] Tonnessen‐Murray CA , Frey WD , Rao SG , et al. Chemotherapy‐induced senescent cancer cells engulf other cells to enhance their survival. J Cell Biol. 2019;218(6):3827‐3844. 31530580 10.1083/jcb.201904051 PMC6829672

[14] Evans TL , Cho BC , Udud K , et al. Cabazitaxel versus topotecan in patients with small‐cell lung cancer with progressive disease during or after first‐line platinum‐based chemotherapy. J Thorac Oncol. 2015;10(8):1221‐1228. 26200278 10.1097/JTO.0000000000000588

[15] Gou D , Wang J , Gao LI , et al. Identification and functional analysis of a novel human KRAB/C2H2 zinc finger gene ZNF300. Biochim Biophys Acta. 2004;1676(2):203‐209. 14746915 10.1016/j.bbaexp.2003.11.011

[16] Cao Y , Li JX , Ji CN , et al. Molecular cloning and characterization of a novel splice variant of human ZNF300 gene, which expressed highly in testis. DNA Seq. 2007;18:312‐315. 17541838 10.1080/10425170701243393

[17] Yu J , Yan FJ , Han CJ , et al. ZNF300 promotes megakaryocytic differentiation. J Exp Hematol. 2017;25:1537‐1543. 10.7534/j.issn.1009-2137.2017.05.046 29070140

[18] Yan F‐J , Wang Y‐J , Yan S‐R , et al. ZNF300 stimulates fatty acid oxidation and alleviates hepatosteatosis through regulating PPARα. Biochem J. 2019;476(2):385‐404. 30568000 10.1042/BCJ20180517

[19] Xue LU , Qiu H , Ma J , et al. ZNF300, a recently identified human transcription factor, activates the human IL‐2Rβ promoter through the overlapping ZNF300/EGR1 binding site. Cell Mol Biol Lett. 2010;15(4):530‐540. 20585888 10.2478/s11658-010-0025-1 PMC6275642

[20] Wang T , Wang X‐G , Xu J‐H , et al. Overexpression of the human ZNF300 gene enhances growth and metastasis of cancer cells through activating NF‐kB pathway. J Cell Mol Med. 2012;16(5):1134‐1145. 21777376 10.1111/j.1582-4934.2011.01388.x PMC4365892

[21] Xu J‐H , Wang T , Wang X‐G , et al. PU.1 can regulate the ZNF300 promoter in APL‐derived promyelocytes HL‐60. Leuk Res. 2010;34(12):1636‐1646. 20471086 10.1016/j.leukres.2010.04.009

[22] Zhao Y , Xue F , Sun J , et al. Genome‐wide methylation profiling of the different stages of hepatitis B virus‐related hepatocellular carcinoma development in plasma cell‐free DNA reveals potential biomarkers for early detection and high‐risk monitoring of hepatocellular carcinoma. Clin Epigenetics. 2014;6:30. 25859288 10.1186/1868-7083-6-30 PMC4391300

[23] Bowden NA , Croft A , Scott RJ . Gene expression profiling in familial adenomatous polyposis adenomas and desmoid disease. Hered Cancer Clin Pract. 2007;5(2):79‐96. 19725988 10.1186/1897-4287-5-2-79 PMC2736996

[24] Chang L , Karin M . Mammalian MAP kinase signalling cascades. Nature. 2001;410(6824):37‐40. 11242034 10.1038/35065000

[25] Al‐Ejeh F , Kumar R , Wiegmans A , et al. Harnessing the complexity of DNA‐damage response pathways to improve cancer treatment outcomes. Oncogene. 2010;29(46):6085‐6098. 20818418 10.1038/onc.2010.407

[26] Lens SM , Voest EE , Medema RH . Shared and separate functions of polo‐like kinases and aurora kinases in cancer. Nat Rev Cancer. 2010;10(12):825‐841. 21102634 10.1038/nrc2964

[27] Prevo R , Pirovano G , Puliyadi R , et al. CDK1 inhibition sensitizes normal cells to DNA damage in a cell cycle dependent manner. Cell Cycle. 2018;17(12):1513‐1523. 30045664 10.1080/15384101.2018.1491236 PMC6132956

[28] Dauch D , Rudalska R , Cossa G , et al. A MYC‐aurora kinase A protein complex represents an actionable drug target in p53‐altered liver cancer. Nat Med. 2016;22(7):744‐753. 27213815 10.1038/nm.4107

[29] Ahmed F , Haass NK . Microenvironment‐driven dynamic heterogeneity and phenotypic plasticity as a mechanism of melanoma therapy resistance. Front Oncol. 2018;8:173. 29881716 10.3389/fonc.2018.00173 PMC5976798

[30] Perego M , Maurer M , Wang JX , et al. A slow‐cycling subpopulation of melanoma cells with highly invasive properties. Oncogene. 2018;37(3):302‐312. 28925403 10.1038/onc.2017.341 PMC5799768

[31] Marie R , Webster MEF , Gretchen M , et al. Paradoxical role for wild‐type p53 in driving therapy resistance in melanoma. Mol Cell. 2020;77:1‐12. 32032511 10.1016/j.molcel.2020.01.005 PMC7441646

[32] Luo H , Yang A , Schulte BA , et al. Resveratrol induces premature senescence in lung cancer cells via ROS‐mediated DNA damage. PLoS ONE. 2013;8(3):e60065. 23533664 10.1371/journal.pone.0060065 PMC3606183

