# Supplementary figures and images for: ZNF300 promotes chemoresistance and aggressive behaviour in non‐small‐cell lung cancer
Source: Cell Prolif. 2020 Oct 19;53(11):e12924. doi: 10.1111/cpr.12924 (PMC7653252; doi:10.1111/cpr.12924)

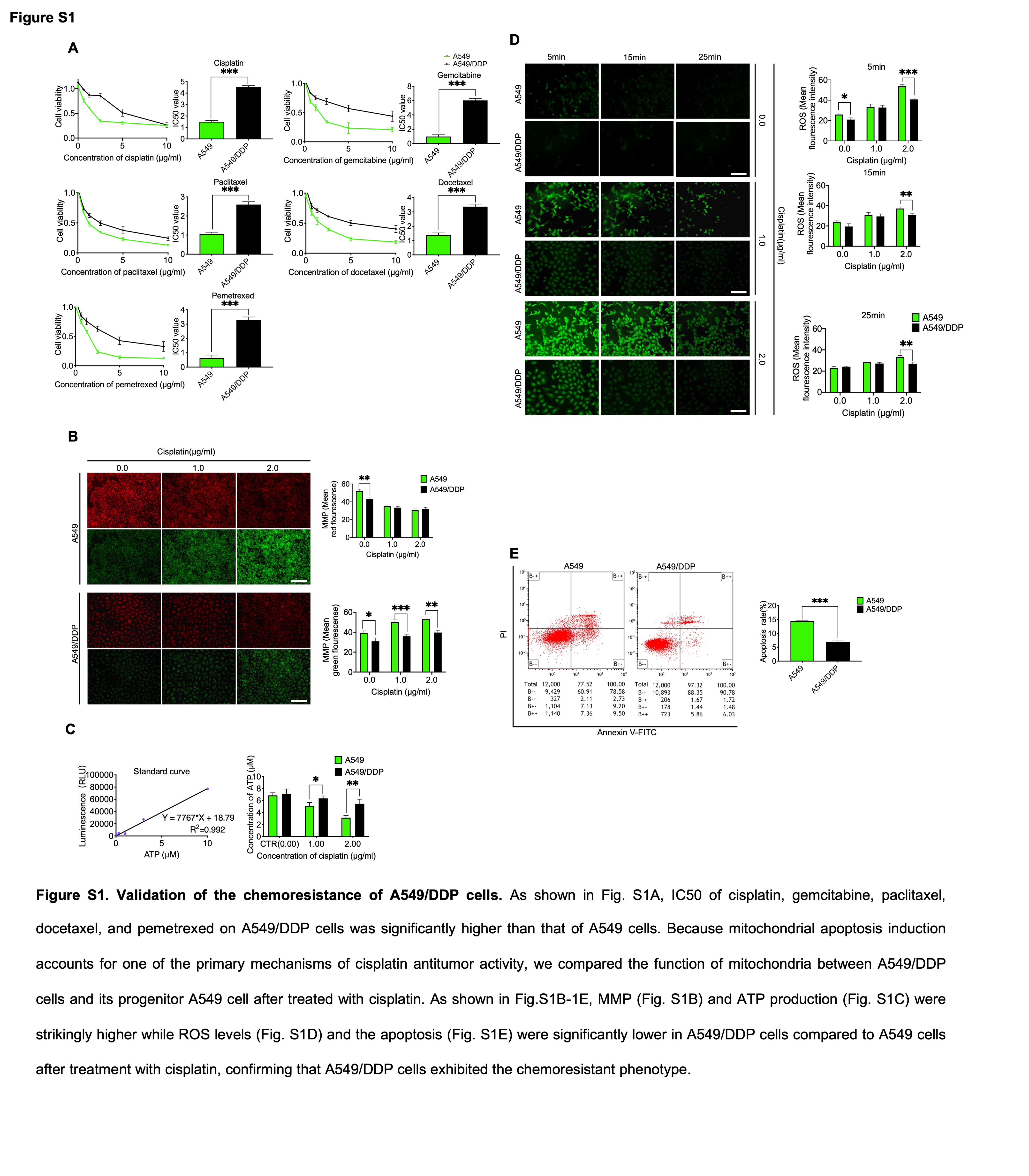

Supplement: Supplementary file 1 — Fig S1 [file CPR-53-e12924-s001.png]

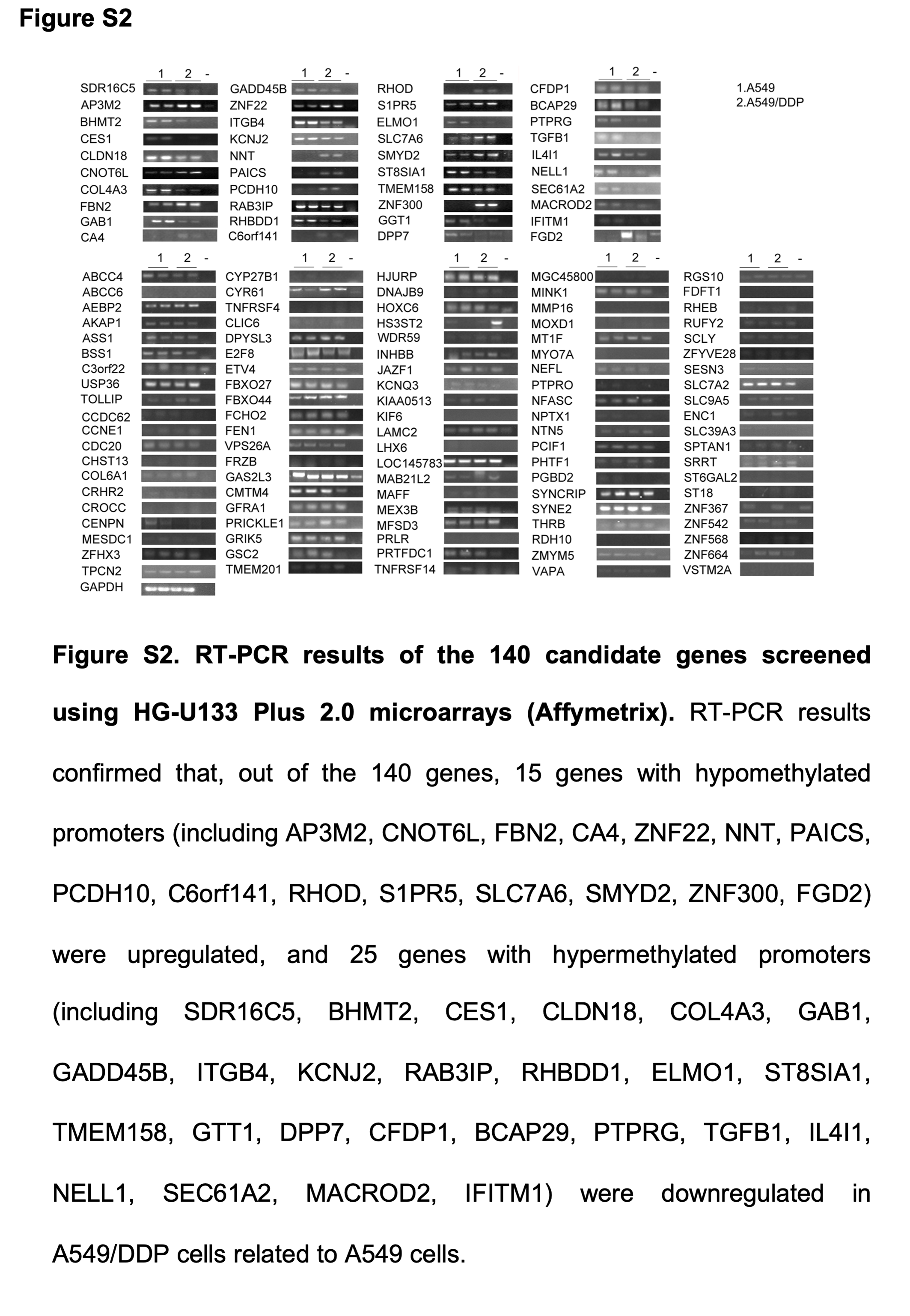

Supplement: Supplementary file 2 — Fig S2 [file CPR-53-e12924-s002.png]

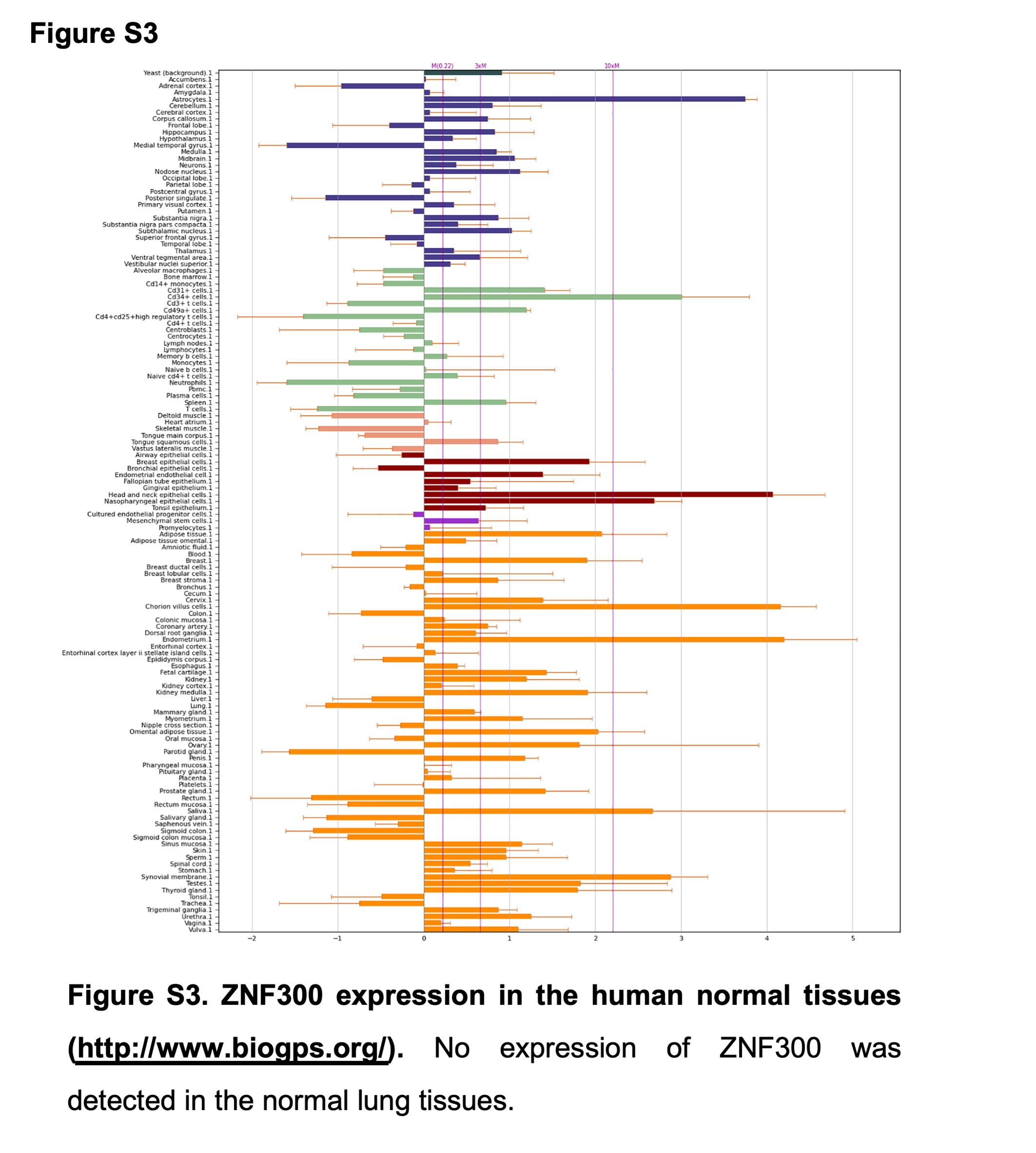

Supplement: Supplementary file 3 — Fig S3 [file CPR-53-e12924-s003.png]

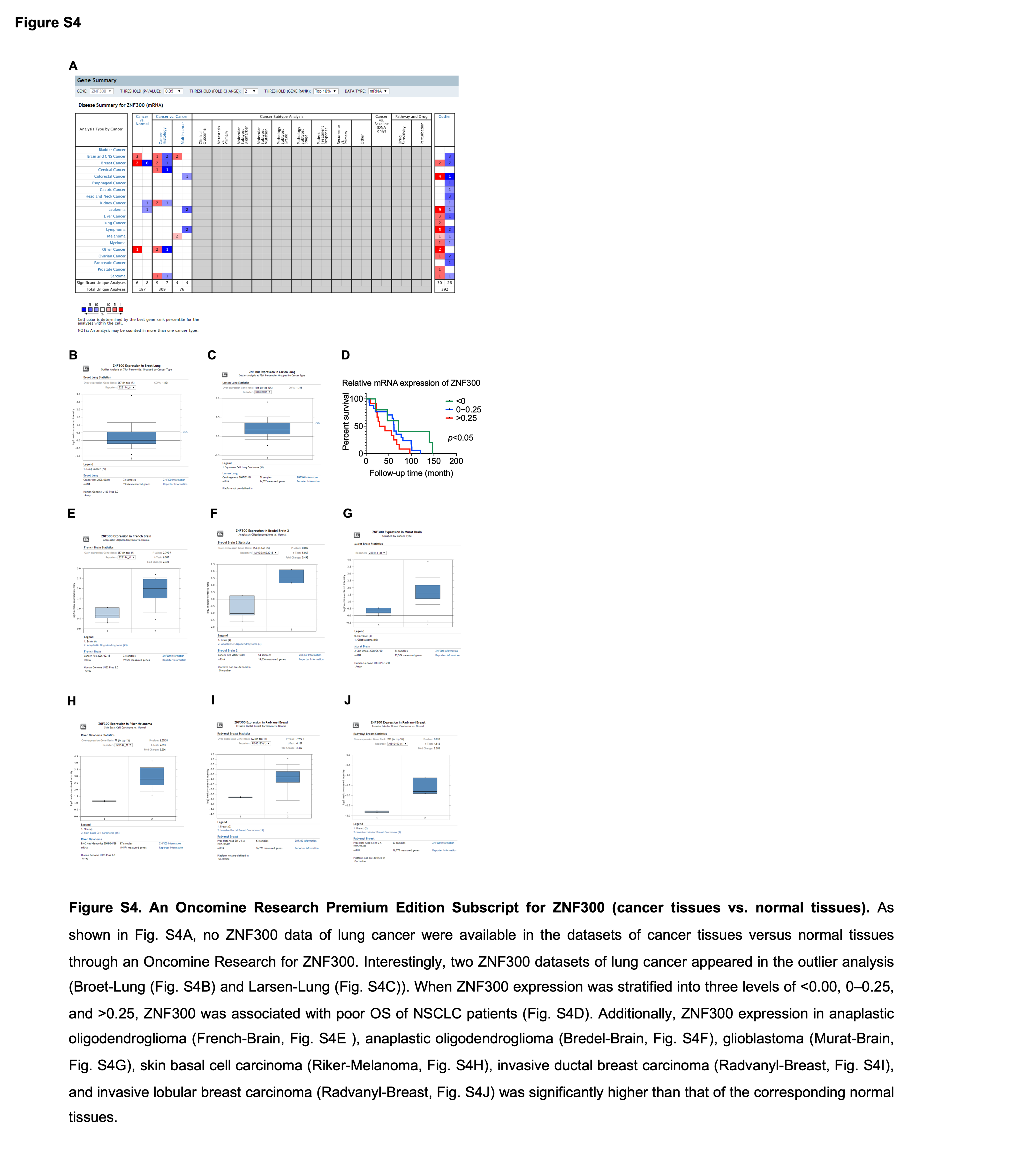

Supplement: Supplementary file 4 — Fig S4 [file CPR-53-e12924-s004.png]

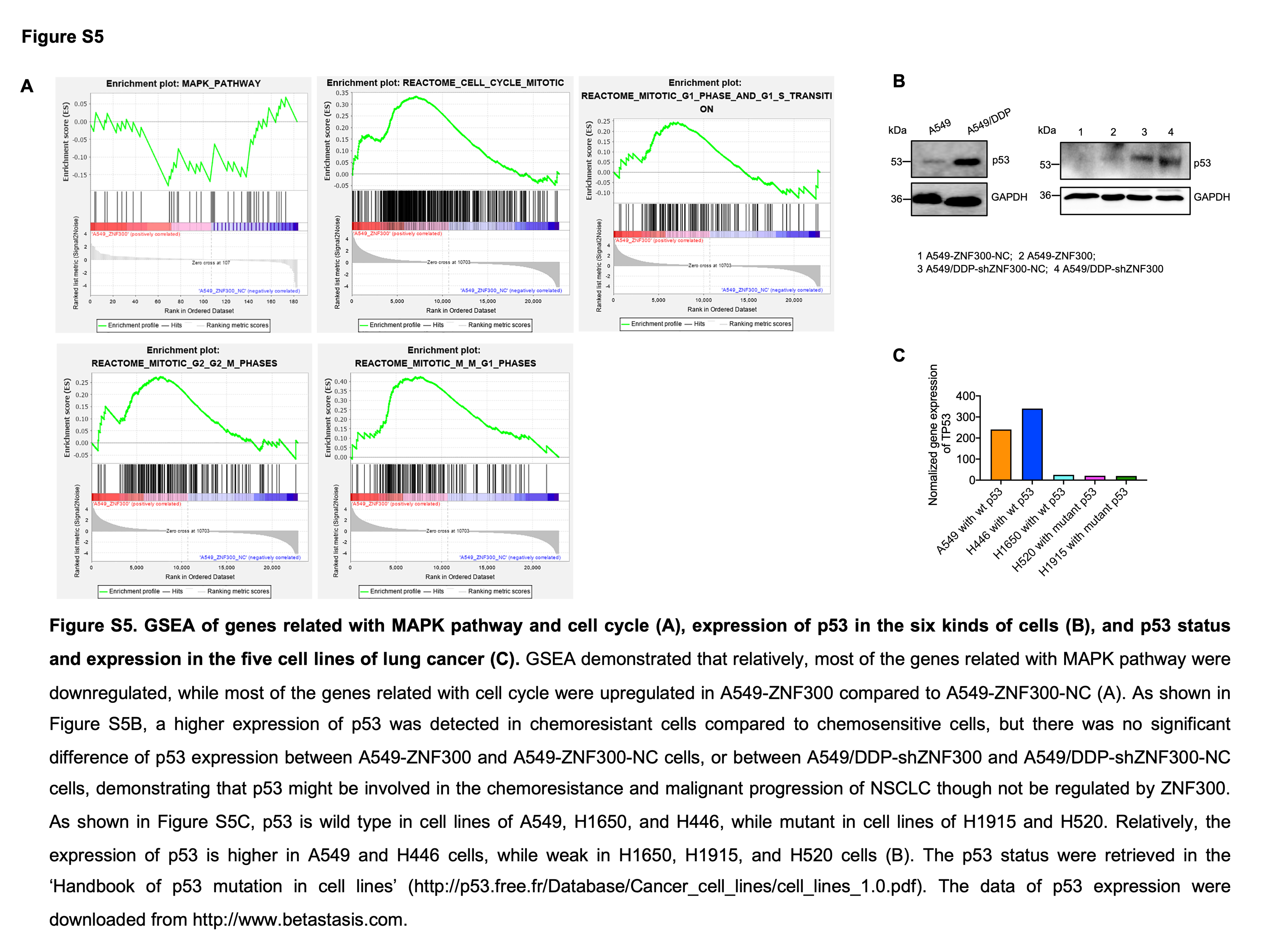

Supplement: Supplementary file 5 — Fig S5 [file CPR-53-e12924-s005.png]
