# Supplementary material for: ZNF300 promotes chemoresistance and aggressive behaviour in non‐small‐cell lung cancer
Source: Cell Prolif. 2020 Oct 19;53(11):e12924. doi: 10.1111/cpr.12924 (PMC7653252; doi:10.1111/cpr.12924)
Supplement: Supplementary file 6 — Table S1 [file CPR-53-e12924-s006.docx]

# **Table S1** Primer sequences of the 140 candidate genes screened using HG-U133 Plus 2.0 microarrays (Affymetrix) and genes related with senescence and SASP for RT-PCR.

| Gene symbol | Forward (5’-3’) | Forward (5’-3’) |
| --- | --- | --- |
| *AP3M2* | TTGAGGCGCAAGAGAGAGCTA | GTCCACCACTCGGTGAAGAAA |
| *BBS1* | CAGGCCCTTGTGTCTATGTGT | GGGTCGATTCGGTCCTCTTT |
| *BCAP29* | GCAGACCTGATGCCTATGAAC | TTGCCAGTTGAGTAATAAGCGT |
| *C6orf141* | CCAGAGAGGTGGTTAGGGACT | CTCGTTGAAAGACAGAAGGGTAG |
| *CA4* | CTGGTGCTACGAGGTTCAAGC | GAAGAAGAAGCGTCCCAGTTT |
| *CCNE1* | ACTCAACGTGCAAGCCTCG | GCTCAAGAAAGTGCTGATCCC |
| *CDC20* | GCACAGTTCGCGTTCGAGA | CTGGATTTGCCAGGAGTTCGG |
| *CENPN* | TGAACTGACAACAATCCTGAAGG | CTTGCACGCTTTTCCTCACAC |
| *CHST13* | CCGGCATTTGGAAACAGAGC | CGGGTCCTGATCCAGGTCAT |
| *CNOT6L* | AGATGGAACCCGAAAGCTACT | TAACCGTGAATGATGCTGACG |
| *CRHR2* | GGTCCCTACTCCTACTGCAAC | CCAAGCATTCTCGATAGGCATTC |
| *DPP7* | GGACCACTTCAACTTCGAGC | GCCCTCGTTCCCAGTGTAG |
| *DPYSL3* | GACCGTCTCCTTATCAAGGGA | GCATCTGGAAGTGAGTATGGAC |
| *ELMO1* | TATTGTCGGCTTTCGCCAAAT | TCAAGCACCTCCTTGTTTTGT |
| *FBXO27* | CCCATCGGACGCAACCTTATT | CCTGCCACTATCCAGCAGTTC |
| *FCHO2* | ATGGTCATGGCGTATTTCGTC | TGGTAGCTCGTTCCCTTACAAA |
| *FEN1* | CACCTGATGGGCATGTTCTAC | CTCGCCTGACTTGAGCTGT |
| *FBN2* | TGGATTTTGTTCCCGTCCTAAC | CAACGTCCACCATTCTGACAT |
| *GAS2L3* | GCTCGGGACAATACCGCAAA | GGCTCAACCCCGTATCTTGAC |
| *GSC2* | CCAGTATCCTGACGTGAGTACG | GCTTCTGGTGTCGCCATTTG |
| *HJURP* | CACAAAGCCATCAAGCATCATC | TCAGAGCAGGGTATGAAGTTCT |
| *HOXC6* | ACAGACCTCAATCGCTCAGGA | AGGGGTAAATCTGGATACTGGC |
| *IL4I1* | GCCAAGACCCCTTCGAGAAAT | CCGATCCTGTTATCTGCCTCC |
| *KIF6* | TCTGCAACTGTACGACATGCC | TGGCCTCTGTTAGAAGATGGC |
| *LAMC2* | TGGAGAACGCTGTGATAGGTG | CAGGAGACCCATTTCGTTGGA |
| *LHX6* | GGGCGCGTCATAAAAAGCAC | TGAACGGGGTGTAGTGGATGT |
| *LOC145783* | TTTCCAGAATCCACGCCTCC | TCACTTTAGGGAGCAGCACG |
| *MESDC1* | CGCCAAGATGTCGGACCAC | TCTCCCTTAAAGCCTGAGATAGC |
| *MEX3B* | GACGCACACGTACATCGTGA | AAGTCGTTCTCGTCTGTGAGC |
| *MGC45800* | GTCGTCCTTGTTCTCCCAGTG | GTAAAACGACGGCCAGT |
| *MMP16* | AGCACTGGAAGACGGTTGG | CTCCGTTCCGCAGACTGTA |
| *MYO7A* | GCAGAACGCAACGCACATC | TCCCGGTAGCGGATAAGCA |
| *NEFL* | ATGAGTTCCTTCAGCTACGAGC | CTGGGCATCAACGATCCAGA |
| *NNT* | GGGGTCCTGTAAGGGTCTAC | ATGCCACTCGCTTCTCATTTT |
| *NPTX1* | AGCTCACATTCCCACTGCG | CACAGCGTAGGAGAAGGGC |
| *ZFYVE28* | TGCGGGACCTGAACACCTA | CATGGCCGAGACGTAGCTG |
| *PAICS* | TTGCAGAAGAATAGCAACTGGTT | CACTGTGGGTCATTATTGGCAT |
| *PCIF1* | CGATGTGATTTCGGACCCTTT | GCTTTCTGGGCTTGTTCTCAG |
| *PRICKLE1* | TTTGCTTGCTTACCAGAGGAAA | ACTGGCAATACCGTACCTCAT |
| *PRTFDC1* | TTGTGGACAGAATTGAGCGG | TCTGAATTTCGGCTGATGTTCTT |
| *PTPRG* | TGACAGAAGGCTACGTTGGG | TCAGGACCATAGGCACCAGAG |
| *PTPRO* | CCCAATGTGGTAGTGATCTCCG | AGCTTTCCATCCCTCTCTAAGG |
| *RHOD* | TCCGAAGAAACGGATTGGAGC | GACGTTGTCATGGAGCCGA |
| *S1PR5* | GCGCACCTGTCCTGTACTC | GTTGGTGAGCGTGTAGATGATG |
| *SCLY* | TGGTGAAACATTTCCACGCAA | CCGGATGGAGTCGTGTTCC |
| *SESN3* | CTGGGAAAATCATGGGTTCTCC | GCATGGTTGTGTCAACATCCT |
| *SLC39A3* | TGCTCCCCGTGAAGATCATC | TCCAAAGGTGTTGCAGAGAGA |
| *SLC7A2* | CCTTATGGCTTTACGGGAACG | CGAGGAGGTAGTACGGCATCA |
| *SLC7A6* | ATGGGATGTCACTGATTGTGTG | AGGACGGCTGGATGATGTAGT |
| *SMYD2* | CTCCAAGCATCTCGGATTCCC | TGCAACATCAGGAAATATCGCTG |
| *ST6GAL2* | TGCGCTGTCGTCATGTCTG | GCGTATGGTGGTTTTATTCCCAA |
| *THRB* | GGCGCAGCACGTTGAAAAAT | CACATCATCATGGTCCAGATGG |
| *TMEM201* | CGCACACGATGGTCAACTG | GATCGGCTTGTTGTAGTCGC |
| *TNFRSF4* | GCAATAGCTCGGACGCAATCT | GAGGGTCCCTGTGAGGTTCT |
| *TOLLIP* | TGGGCCGACTGAACATCAC | GTGGATGACCTTATTCCAGCG |
| *PRLR* | TCTCCACCTACCCTGATTGAC | CGAACCTGGACAAGGTATTTCTG |
| *VPS26A* | TTCAGGAAAGGTAAACCTAGCCT | ATTGGCACCGATGTAAGATTCAT |
| *WDR59* | ACTCGTGTCATCAGCGACTTG | GCAACAGTAGGTTTCCTTGTGT |
| *ZNF22* | AGTGGGGCATGACTATTCGAT | CCAAGAGGGTAAGTGAGTCTTCA |
| *ZNF367* | GGCTTCAGCGACTTCATGGT | GTCGGATTCCATCCTTGAGGT |
| *ZNF568* | GGCTGTTGACCTTACCCAGG | ATCACATCCGGTTTGGTGACT |
| *ZNF664* | TTGCACTTTGAGAGCCCCTT | AGCTTTTCCCTGCCTGTCTT |
| *HS3ST2* | TTTATCCGAGTACACCCGGAC | ATGTTGAAGATGCGTCGAGGA |
| *PHTF1* | GGGAGCCGAGAAAATGGAAAT | TTGCCACATTCTAATCTCTGGTC |
| *SYNCRIP* | CTGGTCTCAATAGAGGTTATGCG | TCCGGTTGGTGGTATAAAATGAC |
| *RUFY2* | GCTGTAGAGAGAGCAAACTTGT | GGGGATAGTCAGAATCCAAAGTG |
| *ST18* | CAAACCACCTAGAGTCCCAAAG | ACACCTGTTCTCACAAGGGATA |
| *ABCC4* | AGCTGAGAATGACGCACAGAA | ATATGGGCTGGATTACTTTGGC |
| *ABCC6* | AGGAGGCCCGAGCTTAGAC | CCTGCCGTATTGGATGCTGT |
| *ASS1* | TCCGTGGTTCTGGCCTACA | GGCTTCCTCGAAGTCTTCCTT |
| *BHMT2* | GATAGACCCGTGGCAGTTACC | AGCTCCATCGTCTTCAAGCTG |
| *C3orf22* | CAAACGACTCGAACACGGTG | AGGAGCCAGACGGTGATGTA |
| *CCDC62* | GGTCACTCACGAAGAAGGTAAAA | CACTAACGTGTTGCTGAGAGTT |
| *CES1* | CACCCAAGATCCCAAGGCG | CACCACGTTTTCATGGGCAG |
| *CFDP1* | TTCGACTCCGAAGACTTCTCT | CCTGGCATCCTCTGATCCAAT |
| *CLDN18* | ACATGCTGGTGACTAACTTCTG | AAATGTGTACCTGGTCTGAACAG |
| *CLIC6* | AGTATCGGAAATTGCCCGTTT | ACATCCGTCTTGACTTCACCAT |
| *CMTM4* | TTCAATCGTACTGGCTGCTTT | CCAGGAATGTGTTCACTGCATA |
| *COL4A3* | AGCAAGGGTTGTGTCTGTAAAG | CAGAAAATCCTGGCAATCCACT |
| *COL6A1* | ACAGTGACGAGGTGGAGATCA | GATAGCGCAGTCGGTGTAGG |
| *CYP27B1* | GGAACCCTGAACAACGTAGTC | AGTCCGAACTTGTAAAATTCCCC |
| *CYR61* | CTCGCCTTAGTCGTCACCC | CGCCGAAGTTGCATTCCAG |
| *DNAJB9* | TCTTAGGTGTGCCAAAATCGG | TGTCAGGGTGGTACTTCATGG |
| *ENC1* | GCCAGCCATCTATCTCATGGA | GGTTACCACACCGTCATTCTG |
| *ETV4* | CAGTGCCTTTACTCCAGTGCC | CTCAGGAAATTCCGTTGCTCT |
| *FBXO44* | AGATTGCGGGTCCAAGTACC | GCCCAGTAATGAGTGTCCACG |
| *FGD2* | CACCTGGACCGACAAGTCTC | CAGCAGCTCGTAACGTGGAA |
| *FRZB* | TGGAACATGACTAAGATGCCCA | ACACAGACTTACAGGGCTTGAT |
| *GAB1* | GATGGTTCGTGTTACGCAGTG | CGCTGTCTGCTACCAAGTAGAA |
| *GADD45B* | TGCTGTGACAACGACATCAAC | GTGAGGGTTCGTGACCAGG |
| *GGT1* | CTGAGCTGATCGAGCACCC | CTCTACGATGCGGTGGTACG |
| *GRIK5* | CCACCGTGAGCCATATCTGTG | CGCGAAGCGAAGGTACTGAA |
| *IFITM1* | CCAAGGTCCACCGTGATTAAC | ACCAGTTCAAGAAGAGGGTGTT |
| *AKAP1* | TGTCTCGGGAGCATGTCTTG | GCCGACTCGATGAACCTACTT |
| *INHBB* | TGCGGGTCAAAGTGTACTTCC | ACTGCACGTCTAGGTTGAGTC |
| *ITGB4* | GCAGCTTCCAAATCACAGAGG | CCAGATCATCGGACATGGAGTT |
| *KCNJ2* | CTGGCTTTCGTCCTGTCATGG | GCCCACGATTGACTGGAACA |
| *KCNQ3* | TGCCGCCACCTTTTCCTTAAT | TGGTAGCATAATACCTCCAGGC |
| *KIAA0513* | CGTGGGCTCGCTAATCGAC | TTCTCACTGTCCGCAGACTCA |
| *MAB21L2* | CACCAGCGAGGTCAAGTTG | GGTCAGCGAGTAGCACTCC |
| *MACROD2* | AGATGACCTTAGAAGAGAGACGC | CCATGATAGAATGCTGTTCAGGG |
| *MFSD3* | ACTGTGGAATGGTGTGGGTG | GAAGACCAAGGCAGTCTGACA |
| *MINK1* | GACGGAACACTTTCATTGGGA | GGCTGTGATTCCTAGAGACCA |
| *MOXD1* | TCTACAGACCAGTCACGACCT | CCTTGAATCGACCACTCAGCA |
| *NELL1* | CCTACCGCATCTCAGCTTCAG | GTCCACGAAACATAGGGCGAG |
| *NFASC* | CACGGCCCTGTCCAATAGG | CTGGAGCGTCAAAGGAGCG |
| *NTN5* | TGCCGGTTCAACTCTGAGC | CTGTTGCCCCAATAGGGTGG |
| *ST8SIA1* | GTGGTATGACGGGGAGTTTTT | GGGAGATTGCATCGCATGACA |
| *SPTAN1* | TACGAGAATGTGAGGACGTGA | CATGAGCAGCCATATCTGTTTGA |
| *PGBD2* | TGGATCTAGGAGGCAGTATGGT | GTACTCACGAACAGTTCCTGTG |
| *RAB3IP* | GGATGAGCCCACAATGGACA | TCCTGCTGTTTCACGAGTCC |
| *RDH10* | CGTGGTGGAGTTCTTCGTGG | TCGTTGCTTTGCGTGTTGATG |
| *RGS10* | AACCGCACCCTCTGATGTTC | GGCTGTAGCTGTCGTACTTCA |
| *RHBDD1* | ATGCAACGGAGATCAAGAGGG | GCAGGAGCTATACAGTGGCTTC |
| *SDR16C5* | CCGGCTGGGATCTGTTCTTG | CTGCAATCGCAGGTATAGGC |
| *SLC9A5* | TTCACCTGTCTCGGAAAGTAACA | GAGTCCAACACAATAGGAGGC |
| *SYNE2* | GTGGTCTCTGTCAACGTGAGC | GAGCGACTGTCGTAAGCCC |
| *TMEM158* | CTGAACCGTAAGCCCATTGAG | CGCTCCACACCACGATGAC |
| *TNFRSF14* | GTGCAGTCCAGGTTATCGTGT | CACTTGCTTAGGCCATTGAGG |
| *USP36* | CACCACCTCTAGCCAACTACC | GGCGATCTTTTTCAGGTCTCG |
| *VSTM2A* | TGATGCCAACTACGGGGAG | CGTTCTTGCGGGGCTTCATA |
| *ZFHX3* | CAAGTTCACGACGGACAACCT | GCTTGCACTGGTATGAGTCCC |
| *ZMYM5* | AGAGTTGACTGAACAGACTCCT | GACCAAATGAATCCCCTATGTCC |
| *AEBP2* | CCTTTCAAGTGTGTTGTTGGTG | GTAATGAGCGTCGTCTTTTGTTC |
| *TGFB1* | CTAATGGTGGAAACCCACAACG | TATCGCCAGGAATTGTTGCTG |
| *CROCC* | TGAATGCCATGCTCCGAGAAC | CACCTTTCGTATGTCCTCACTC |
| *E2F8* | CCTGAGATCCGCAACAGAGAT | AGATGTCATTATTCACAGCAGGG |
| *FDFT1* | GCAACGCAGTGTGCATATTTT | CGCCAGTCTGGTTGGTAAAGG |
| *GFRA1* | GACTCCTGCAAGACGAATTACA | GCTGCTGACAGACCTTGACT |
| *JAZF1* | ATCACGCTAAGAATGGTCACAG | TCTTCCTGATAATCTCAGCCGA |
| *MT1F* | CCCCCTCCCCTGACTATCAA | AGGAGCAGCAGCTCTTCTTG |
| *PCDH10* | CCAACTCAAGGACCCCTTACT | CGCGGTCTATTTTCTCGTTCA |
| *RHEB* | TTGTGGACTCCTACGATCCAA | GGCTGTGTCTACAAGTTGAAGAT |
| *VAPA* | GAGATGCGTATTTGAAATGCCC | TCTCTCAGGTGCCGATTTTCT |
| *MAFF* | TCACCGCATGACTGGGTTTT | CTGAGTTTTGTTGTGGGGCG |
| *SEC61A2* | TTTATTGCCACCAACATCTGTGA | CCCGTAAAGCTCGGACTTTGT |
| *SRRT* | AAGGCGCAGGGACAAGTTC | CGCCGATTCCGGTCATAGTC |
| *TPCN2* | TCCCTGAGTCTCGTGTGTCAT | AGCATTCCGAACATGGTGAAG |
| *ZNF542* | TGTCTTCCCTGATTGAACATTGG | AGAGTGAACTGCATCACACGA |
| *GAPDH* | CAGCCTCAAGATCATCAGCA | TGTGGTCATGAGTCCTTCCA |
| *ZNF300* | CACCTGGTCTCAATGGGGTAT | TGTGAAGGTTACTCTTCCTGTCT |
| *CDKN2A* | TCCTTCCTCCGCGATACAAC | CCGCGCTCCTGAAAATCAAG |
| *CDKN1A* | GACATGTGCACGGAAGGACT | GGGCAGGGTGACAAGAATGT |
| *IL6* | ACTCACCTCTTCAGAACGAATTG | CCATCTTTGGAAGGTTCAGGTTG |
| *IL8* | ACTGAGAGTGATTGAGAGTGGAC | AACCCTCTGCACCCAGTTTTC |
| *CXCL10* | GTGGCATTCAAGGAGTACCTC | TGATGGCCTTCGATTCTGGATT |
| *MMP2* | GATACCCCTTTGACGGTAAGGA | CCTTCTCCCAAGGTCCATAGC |
| *MMP9* | GGGACGCAGACATCGTCATC | TCGTCATCGTCGAAATGGGC |
| *ACTB* | CATGTACGTTGCTATCCAGGC | CTCCTTAATGTCACGCACGAT |
