# Supplementary material for: ZNF300 promotes chemoresistance and aggressive behaviour in non‐small‐cell lung cancer
Source: Cell Prolif. 2020 Oct 19;53(11):e12924. doi: 10.1111/cpr.12924 (PMC7653252; doi:10.1111/cpr.12924)
Supplement: Supplementary file 7 — Table S2 [file CPR-53-e12924-s007.docx]

**Table S2** Summarization of the genes related with growth, proliferation, differentiation, and cell cycle in the expression profiles based on the keywords of proliferation, growth, differentiation, and cell cycle (A549-ZNF300 vs. A549-ZNF300-NC)

| Sources | Keywords | Gene symbols | Foldchanges | Gene symbols | Foldchanges |
| --- | --- | --- | --- | --- | --- |
| Gene.title | Growth differentiation | GDF1 | 0.67 | GDF2 | 0.14 |
|  |  | GDF10 | 0.51 | GDF3 | 3.05 |
|  |  | GDF11 | 1.97 | GDF5 | 0.79 |
|  |  | GDF15 | 0.37 | GDF9 | 0.40 |
| Pathway | MAPK | AGTRAP | 1.21 | MAP3K12 | 1.19 |
|  |  | ARAF | 1.44 | MAP3K2 | 1.10 |
|  |  | BRAF | 0.68 | MAP3K3 | 1.68 |
|  |  | CSDE1 | 0.97 | MAPK1 | 0.86 |
|  |  | ELK1 | 0.74 | MAPK10 | 0.63 |
|  |  | HDAC10 | 0.66 | MAPK12 | 0.58 |
|  |  | HRAS | 2.75 | MAPK14 | 0.70 |
|  |  | JUN | 0.18 | MAPK3 | 0.54 |
|  |  | KIAA1549 | 1.82 | MBP | 0.65 |
|  |  | KRAS | 0.36 | METAP2 | 1.75 |
|  |  | LIMD2 | 0.65 | MKRN2 | 1.39 |
|  |  | MAP2 | 0.87 | NRAS | 1.49 |
|  |  | MAP2K1 | 1.53 | PLCB3 | 0.65 |
|  |  | MAP2K2 | 0.61 | RAF1 | 0.83 |
|  |  | MAP2K3 | 0.46 | RASA3 | 0.47 |
|  |  | MAP2K4 | 1.15 | RRAS | 0.51 |
|  |  | MAP2K6 | 37.16 | SIPA1 | 1.38 |
|  |  | MAP2K7 | 0.77 | TIMP1 | 0.51 |
|  |  | MAP3K1 | 0.39 |  |  |
|  | Cell_cycle | ABL1 | 0.92 | GSK3B | 0.55 |
|  |  | ATF6B | 1.45 | HABP4 | 0.45 |
|  |  | ATF6B/TNXB | 2.63 | HDAC1 | 0.66 |
|  |  | ATM | 2.46 | HDAC2 | 1.67 |
|  |  | BUB1 | 3.33 | HDAC3 | 0.68 |
|  |  | BUB1B | 5.00 | HDAC4 | 1.69 |
|  |  | BUB3 | 1.49 | HDAC5 | 0.47 |
|  |  | CCNA1 | 0.02 | HDAC6 | 25.00 |
|  |  | CCNA2 | 4.00 | HDAC7 | 0.76 |
|  |  | CCNB1 | 4.76 | HDAC8 | 1.67 |
|  |  | CCNB2 | 4.76 | MAD1L1 | 2.22 |
|  |  | CCNB3 | 4.00 | MAD2L1 | 3.70 |
|  |  | CCND1 | 0.79 | MAD2L2 | 0.71 |
|  |  | CCND2 | 0.29 | MCM2 | 1.61 |
|  |  | CCND3 | 4.76 | MCM3 | 1.85 |
|  |  | CCNE1 | 1.08 | MCM4 | 3.85 |
|  |  | CCNE2 | 2.38 | MCM5 | 2.33 |
|  |  | CCNG2 | 0.53 | MCM6 | 1.49 |
|  |  | CCNH | 1.10 | MCM7 | 2.70 |
|  |  | CDAN1 | 1.19 | MDM2 | 0.07 |
|  |  | CDC14A | 5.88 | MNAT1 | 0.79 |
|  |  | CDC14B | 3.13 | MPL | 10.00 |
|  |  | CDC20 | 2.50 | MYC | 2.18 |
|  |  | CDC25A | 4.35 | MYT1 | 0.16 |
|  |  | CDC25B | 4.00 | NACA | 0.86 |
|  |  | CDC25C | 2.94 | NSUN7 | 11.11 |
|  |  | CDC45 | 2.94 | ORC1 | 2.04 |
|  |  | CDC6 | 3.57 | ORC2 | 0.66 |
|  |  | CDC7 | 1.28 | ORC3 | 0.68 |
|  |  | CDH1 | 50.00 | ORC4 | 0.71 |
|  |  | CDK1 | 3.45 | ORC5 | 2.63 |
|  |  | CDK2 | 2.04 | ORC6 | 1.92 |
|  |  | CDK4 | 1.27 | PCMTD2 | 0.38 |
|  |  | CDK7 | 0.77 | PCNA | 0.42 |
|  |  | CDKN1A | 0.52 | PLK1 | 2.78 |
|  |  | CDKN1B | 1.05 | POLA2 | 2.78 |
|  |  | CDKN1C | 0.15 | POLE | 1.47 |
|  |  | CDKN2A | 1.47 | POLE2 | 2.94 |
|  |  | CDKN2B | 14.86 | PRIM1 | 1.69 |
|  |  | CDKN2C | 2.17 | PRIM2 | 1.28 |
|  |  | CDKN2D | 0.88 | PRIM2/PRIM2B | 9.43 |
|  |  | CGB/1/5/7/8 | 7.14 | PRKDC | 1.52 |
|  |  | CGB1/LHB | 1.16 | PTPRA | 0.78 |
|  |  | CHEK1 | 1.67 | PTTG1 | 2.78 |
|  |  | CHEK2 | 1.82 | RB1 | 0.74 |
|  |  | CREB3 | 1.39 | RBL1 | 2.44 |
|  |  | CREB3L1 | 1.85 | RPA1 | 1.82 |
|  |  | CREB3L4 | 1.04 | RPA2 | 1.27 |
|  |  | DBF4 | 3.57 | RPA3 | 2.50 |
|  |  | DTX4 | 1.79 | RPL31/TBC1D8 | 1.01 |
|  |  | E2F1 | 1.92 | SKP2 | 2.22 |
|  |  | E2F2 | 5.26 | SMAD4 | 1.64 |
|  |  | E2F3 | 0.68 | SMC1A | 1.89 |
|  |  | E2F4 | 0.81 | TBC1D8 | 0.79 |
|  |  | E2F5 | 0.84 | TFDP1 | 1.85 |
|  |  | E2F6 | 1.04 | TFDP2 | 0.51 |
|  |  | EP300 | 1.18 | TGFB1 | 0.45 |
|  |  | ESPL1 | 2.04 | TP53 | 1.27 |
|  |  | GADD45A | 0.31 | VPS16 | 0.75 |
|  |  | GBA2 | 1.28 | WEE1 | 0.88 |
