# Supplementary material for: ZNF300 promotes chemoresistance and aggressive behaviour in non‐small‐cell lung cancer
Source: Cell Prolif. 2020 Oct 19;53(11):e12924. doi: 10.1111/cpr.12924 (PMC7653252; doi:10.1111/cpr.12924)
Supplement: Supplementary file 8 — Doc S1 [file CPR-53-e12924-s008.docx]

**Supplemental materials and Experimental Procedures**

**Chemicals and kits**

ATP Assay Kit (Cat. No: #S0026), Mitochondrial membrane potential (MMP) assay kit with JC-1 (Cat. No: #C2006), Intracellular Reactive Oxygen Species (ROS) Assay Kit (Cat. No: #S0033), DAPI Staining Solution (Cat. No: #C1005), Antifade Mounting Medium (Cat. No: #P0126), Genomic DNA Mini Preparation Kit (Cat. #D0063), RIPA lysis solution (Cat. No: #P0013B), BCA protein assay kit (Cat. No: #P0010), and Senescence β-Galactosidase (SA-β-GAL) Staining Kit (Cat. No: #C0602Z) were purchased from Beyotime Biotech Co., Ltd (Shanghai, China). Cell Counting Kit-8 (CCK-8) (Cat. No: #A311-01) and Annexin V-PE/7-AAD Apoptosis Detection Kit (Cat. No: #A213-01) were obtained from Vazyme Biotech Co., Ltd (Nanjing, China). Cell Light EdU DNA imaging Kit (Cat. No: #C10310-1) was procured from RiboBio Biotech Co., Ltd (Guangzhou, China). 3’IVT Express Kit was obtained from Affymetrix Inc., CA, USA (Cat.No: #902416). All other main reagents and kits used have been listed as follows: Matrigel (Cat. No: #354234, BD Bioscience, USA), Propidium Iodide (PI) solution (Bestio, Shanghai, China), EZ DNA Methylation Gold™ Kit (Cat. #D5006, ZymoResearch, USA), ZymoTaq™ DNA polymerase (Cat. #E2001, CA, USA), TA cloning Kit (Cat. #K2020-20, Invitrogen, USA), poly-2-hydroxyethyl methacrylate (poly-HEMA, Sigma, USA), PrimeScript™ II first Strand cDNA Synthesis Kit (Cat. #6210B, Takara Bio., Dalian, China), SYBR GREEN MIX kit (Promega, Madison, WI, USA), PVDF membranes with a pore size of 0.45 µm (Millipore, Billerica, MA, USA), Enhanced chemiluminescence solution (ECL, Sangon Biotech, Shanghai, China), TRIzol reagent (Cat. #15596026, Invitrogen, USA), AZD6244 (Lot# HY-50706, 10 mM in DMSO, MCE, New Jersey, USA), Hesperetin (Lot# HY-N0168, 10 mM in DMSO, MCE, New Jersey, USA), all-trans retinoic acid (Lot# WXBC9849V, Sigma, USA), and icariin (Lot# B21576, Yuanye, Shanghai, China).

**Antibodies**

Rabbit Anti-ZNF300 polyclonal antibodies (66 kDa, Lot#ab202965) were from Abcam Plc, Cambridge, UK. Mouse anti-Beta-actin monoclonal (43 kDa, Cat.No:#60008-1-Ig), mouse anti-GAPDH monoclonal (36 kDa, Cat.No:#60004-1-Ig), goat anti-rabbit IgG (H+L) (Cat.No:#10285-1-AP), and goat anti-mouse IgG (H+L) (Cat.No:#10285-1-AP) were from Proteintech Group Inc.，Chicago，IL，USA. Alexa Fluor 555-labeled donkey anti-rabbit IgG (H+L) was from Beyotime Biotechnology, Shanghai, China (Cat. No: #A0453). Other antibodies are described as follows: rabbit anti-ATF3 antibody (bs-23807R), rabbit anti-ATF5 antibody (bs-12542R), rabbit anti-phospho-STAT3 (Tyr705) antibody (bs-1658R), rabbit anti-STAT3 antibody (bs-1141R), rabbit anti-Phospho-Stat4 (Tyr693) antibody (bs-3430R), rabbit anti-STAT4 antibody (bs-10023R), PCNA (bs-0754R), CD61 (bs-0342R), CD235a (bs-10017R), p15 (bs-4269R), p27 (bs-0742R), E2F3(bs-1722R) and  PU.1/Spi1 (bs-19594R)  were purchased from Beijing Biosynthesis Bio Technology CO., LTD., Beijing, China. Phospho-p38 MAPK (Thr180/Tyr182) antibody (AF4001), p38 MAPK antibody-C-terminal (AF6456), p44/42 MAPK (Erk1/2) antibody (BF0412), and Phospho-ERK1/2 (Thr202/Tyr204) antibody (AF1015) were purchased from Affinity Biosciences, OH, USA. Oct4 (F0708), Nanog (G0330), and Myc-tag (G0626) were purchased from Cusabio Technology LLC, Wuhan, China. CDK1 antibody (MA5-11472), WEE1 antibody (PA5-67588), Aurora A (AURKA) antibody (PA1-26351), and PLK1 antibody (37-7000) were purchased from Thermo Fisher Scientific, Waltham, MA, USA.

**Recombinant lentiviral vector**

The sequences of ZNF300-shRNA oligonucleotides were as following: shZNF300-1661 (5'-GCTGA TTATACACCACAGAGC-3'), shZNF300-2777 (5'-GGTTGATCAGATTCAGTAAAG-3'), shZNF300-2659 (5'-GACGTGGAAGAAAGCTTAAGT-3'), and shZNF300-2522 (5'-GATCAGCACAGCAAGTTAAGC-3'). The LV3 and LV5 vectors containing a random DNA sequence (5’-TTCTCCGAACGTGTCACGT-3') were used as vector controls (LV3-NC and LV5-NC, respectively). All of the recombinant lentiviral vector constructs had green fluorescent protein (GFP) for detection and puromycin resistance for selection. The shRNA oligonucleotides and the full-length sequence coding for human ZNF300 were designed and synthesized by GenePharma (Shanghai GenePharma Co., Ltd., Shanghai, China). For co-culture, the mCherry-labeled LV8 and mCherry-labeled LV10 lentiviral vectors, which contained a random DNA sequence, as mentioned above, were used to transfect the corresponding cells.

**Detection of MMP, ATP, and ROS**

For MMP assay, cells were plated in 6-well plates and allowed to adhere overnight before treatment with cisplatin (0.0, 1.0, and 2.0 µg/mL, respectively). After 24 h of treatment, cells were incubated with 5 µmol/L fluorescent dye JC–1 for 30 min at 37 °C in the dark, washed with PBS to remove the excess dye, and then observed using fluorescence microscopy (Olympus BX51, Japan).

To measure ATP production, after seeding into 96-well plates (5×10^3^ cells/well) for 24 h, cells were treated with cisplatin (0.0, 1.0, and 2.0 µg/mL, respectively) for 48 h. Then ATP production was detected using a commercial ATP assay kit following the manufacturer’s protocol. The luminescence was measured using a microplate reader (Beckman Coulter SP-Max2300A2).

The levels of ROS were detected with an oxidation sensitive fluorescent dye DCFH-DA. Briefly, 5×10^5^ cells were plated in 12-well plates and allowed to adhere overnight. After that, 1 mL serum-free medium with 1 µL DCFH-DA (10 mM) was added to each well and incubated at 37 °C for 20 min. The cells were then incubated with cisplatin (0.0, 1.0, and 2.0 µg/mL, respectively), and the DCF fluorescence pictures were captured after every 5 min by fluorescence microscopy (Olympus BX51, Japan).

**RT-PCR**

After total RNA concentration was estimated using Nanodrop-2000, two mg aliquots were reverse transcribed using the PrimeScript™ RT reagent Kit with gDNA Eraser (Perfect Real Time). For RT-PCR, SYBR GREEN MIX kit was used according to the manufacturer’s protocol, and GAPDH was used to normalize the level of gene expression. The relative quantification was determined by gray values within the reference for RT-PCR.

**Drug sensitivity assay in vitro by CCK-8 or RTCA**

Briefly, the different concentrations of cisplatin, gemcitabine, paclitaxel, docetaxel, and pemetrexed were added into 96-well plates seeded with 5×10^3^ cells and treated for 48 h. Then cells of each well were incubated with 100 µL fresh culture medium containing 10 µL CCK-8 solution for 1.5 h. The absorbance was measured at a wavelength of 450 nm. Cells incubated without chemotherapeutic drugs were considered as negative controls. The 50% inhibitory concentrations (IC50) were calculated using GraphPad Prism 5.0 software.

For real-time cell analysis (RTCA, xCELLigence RTCA DP version, ACEA Biosciences, USA), after the well baseline of the E-Plate 16-plates were measured with 50 µL medium containing 10% FBS, cells were seeded at a density of 5×10^3^/well in the same medium to a final volume of 150 µL and incubated for 30 min at room temperature in the laminar flow cabinet. Then the plates were placed in the humidified incubator with 37 °C and 5% CO2 overnight. The next day, the medium was replaced with fresh medium containing gradient concentration of the corresponding drug, and the signals were recorded every 30 min until the end of the experiment (up to 100 h). The cell index data were analyzed via RTCA Software 2.0 to calculate IC50 value.

**Methylation analysis**

DNA samples (500 µg) were extracted using the Genomic DNA Mini Preparation Kit and treated with bisulfite using the EZ DNA Methylation Gold™ Kit. Bisulfite-converted genomic DNA was amplified using ZymoTaq™ DNA polymerase. The BSP specific primers (ZNF300-BSP-F: TAAAAGTGTGTTTTTTAATGTTTTTTT and ZNF300-BSP-R: TAACAAACTCTCCTACTTCCTATCC) were designed according to the location of ZNF300 CpG islands. PCR products were inserted into the pTG19-T vector and ten clones per sample were sequenced.

**Western blotting**

The concentrations of total cellular protein extracted using RIPA lysis solution were determined using a BCA protein assay kit. Protein samples were resolved in SDS denatured polyacrylamide gel and transferred onto PVDF membranes. After blocking in 5% skim milk, membranes were incubated with mouse/rabbit anti-human antibodies overnight at 4 °C. After washed in TBST, membranes were incubated with a secondary anti-mouse/rabbit antibody for 1 h. ECL solution was added onto the membranes and protein expression was quantified using the Laboratory Work Image Acquisition and Analysis Software (ChemiDoc MP, BIO-RAD, CA, USA). β-Actin or GAPDH was used as a loading control.

**Cell cycle, cell apoptosis, and Anoikis resistance assay**

For cell cycle assay, after immobilized in pre-chilled 70% ethyl alcohol, washed with PBS, resuspended in 400 µL PI solution, cells (5–10×10^6^) were acquired on a flow cytometer (Beckman Coulter, Gallios, California, USA). Cells in G1, S, and G2 phases were analyzed by Flow Job Software. To detect apoptosis, after treated with chemotherapeutic agents for 48 h, cells were collected and analyzed as cell cycle assay. For cells transfected with GFP lentivirus vectors, PI staining was replaced by Annexin V-PE/7-AAD staining. For anoikis resistance assay, cells were seeded in the 6-well plates pre-coated with poly-HEMA and cultured for 48 h (5×10^5^ cells per well). Then the suspended cell clones were captured under a light microscope (Olympus BX51, Japan) in 100× and collected to detect apoptotic proportion by flow cytometry.

**5-ethynyl-2’-deoxyuridine assay**

After cells (5×10^3^ cells per well) were seeded into 96-well plates and allowed to adhere overnight, EdU was added and incubated for an additional 2 h. Then cells were stained with premixed Apollo and Hoechst. Images were acquired and analyzed using High Content Imaging Pathway 855 (BD, USA). The fraction of EdU positive cells was calculated using the formula: (EdU add-in cells/Hoechst stained cells) × 100%.

**Migration and invasion assay**

Cellular migration was assessed by wound healing assay. Cells were photographed at 0 and 20 h, respectively, under a light microscope (Olympus BX51, Japan). The invasion was evaluated using the modified Boyden chambers with Matrigel pre-coated filter inserts (pore size of 8 µm, Corning Inc., Life Science). Cells that migrated into the reverse side of the Transwell membrane were fixed with 4% triformol, stained with 0.4% crystal violet, and then counted under a light microscope at 100× magnification. An average of five visual fields was examined.

**Senescence β-Galactosidase staining**

Cells (1×10^4^/well) were seeded on 24-well plate and cultured in medium with corresponding drugs, separately or combined, for 48 h. The concentrations of cisplatin, ATRA, and ICA were 2, 5, and 20 µg/ml (the IC_50_ concentration of the three chemicals on A549 cells), respectively. After being fixed in β-galactosidase staining fixative at room temperature and washed with PBS for three times, cells were incubated in staining solution prepared according to the formula at 37°C overnight (around 8 h). Cells were observed and captured by fluorescence microscopy the next day (Olympus BX51, Japan). The percentage of SA-β-GAL positive cells (%) was calculated as the number of positively stained cells/total number of cells × 100%.
